# Supplementary figures and images for: Shared risk factors for malaria and schistosomiasis co-infection: A systematic review and meta-analysis
Source: PLoS Negl Trop Dis. 2026 Jun 15;20(6):e0014369. doi: 10.1371/journal.pntd.0014369 (PMC13268186; doi:10.1371/journal.pntd.0014369)

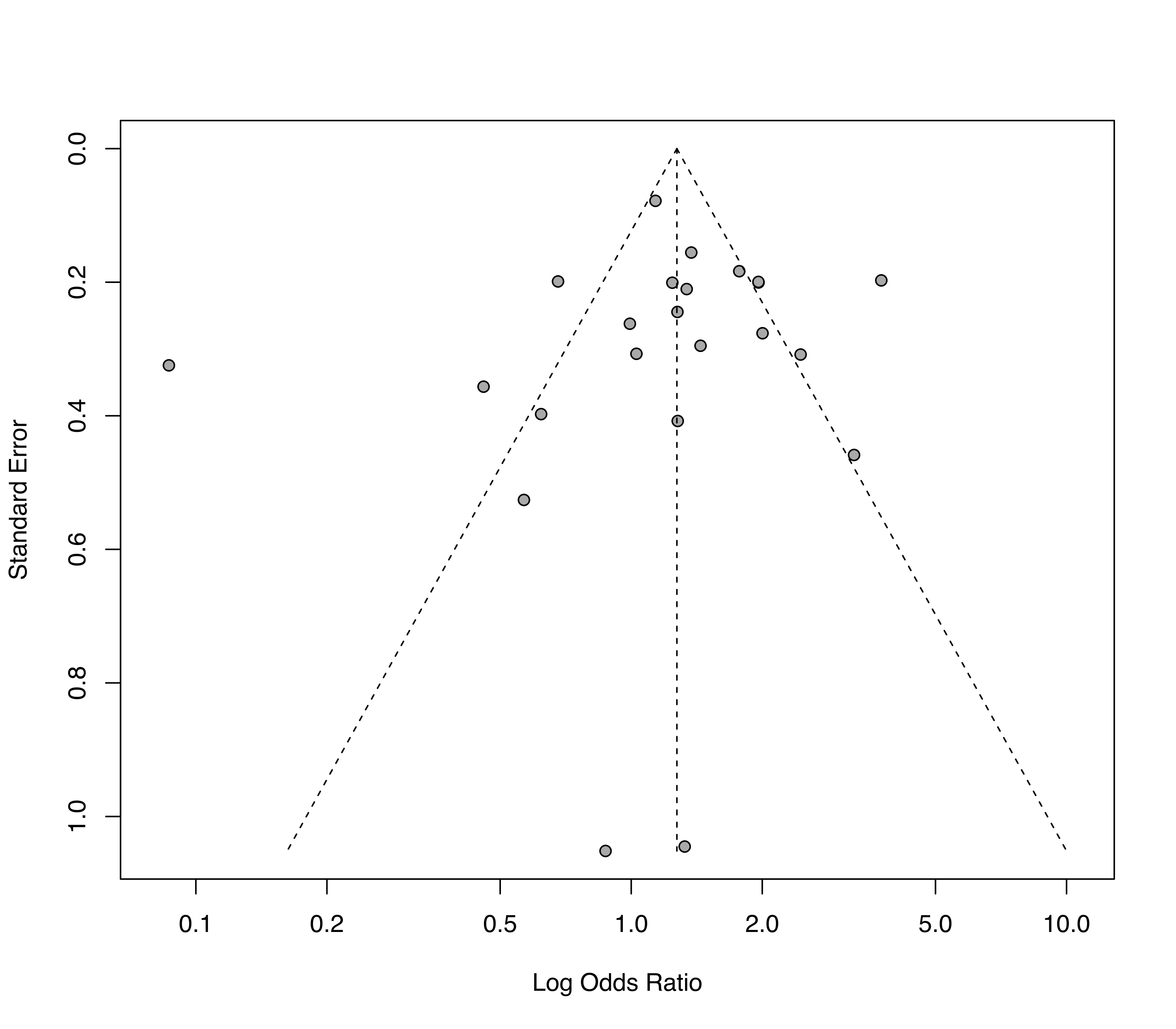

Supplement: S1 Fig — (TIFF) [file pntd.0014369.s001.tiff]

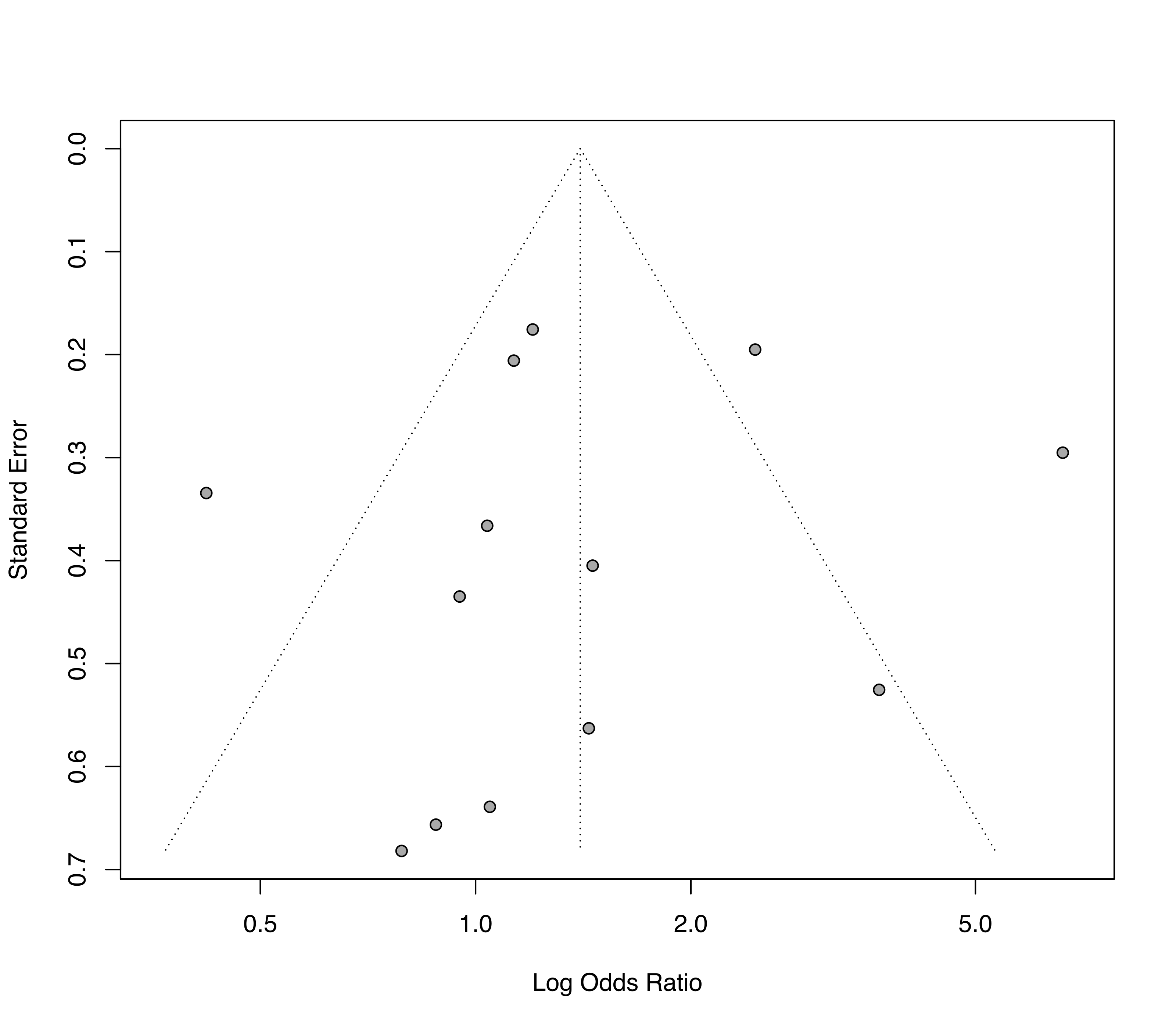

Supplement: S2 Fig — (TIFF) [file pntd.0014369.s002.tiff]

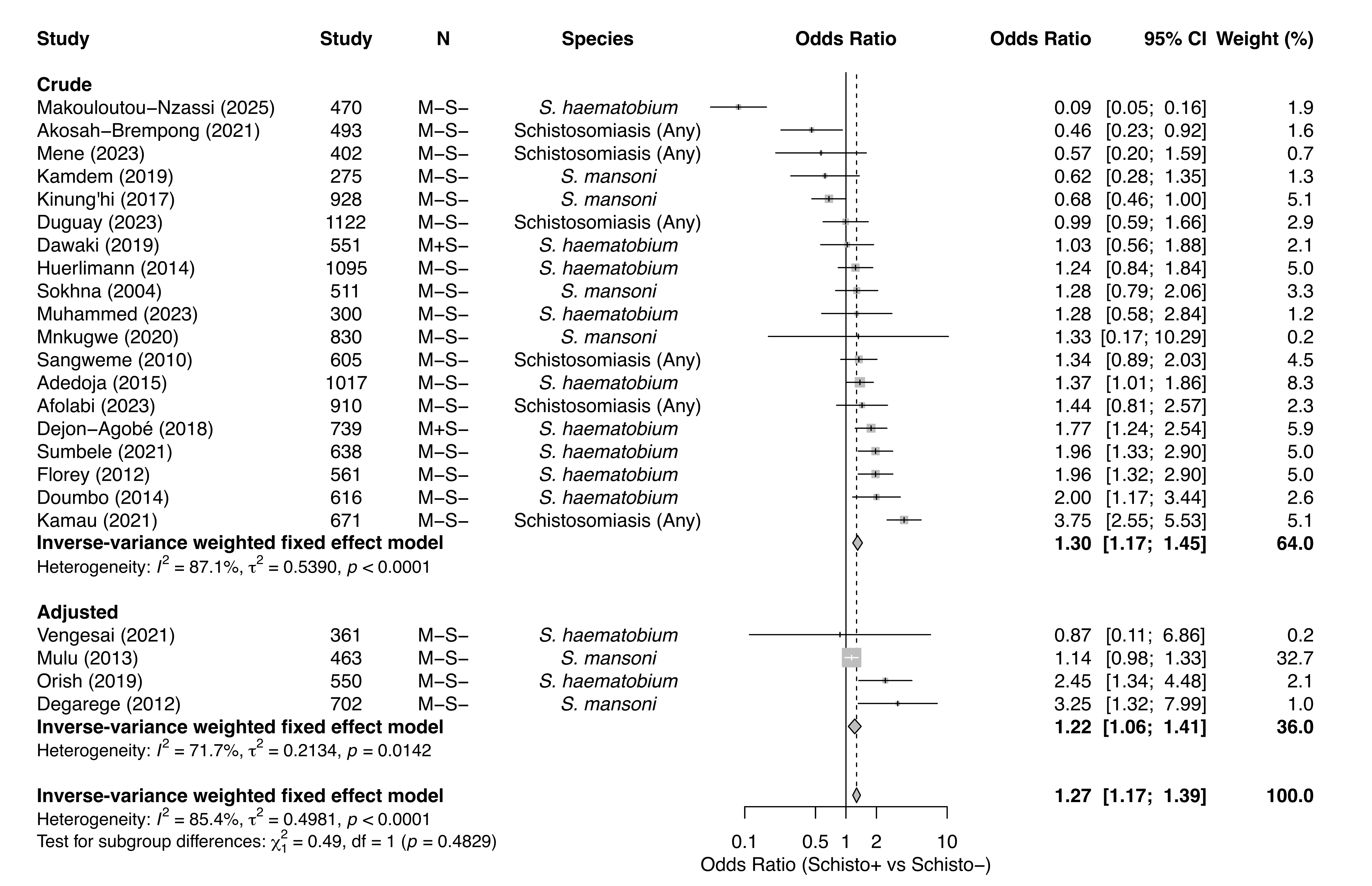

Supplement: S3 Fig — (TIFF) [file pntd.0014369.s003.tiff]

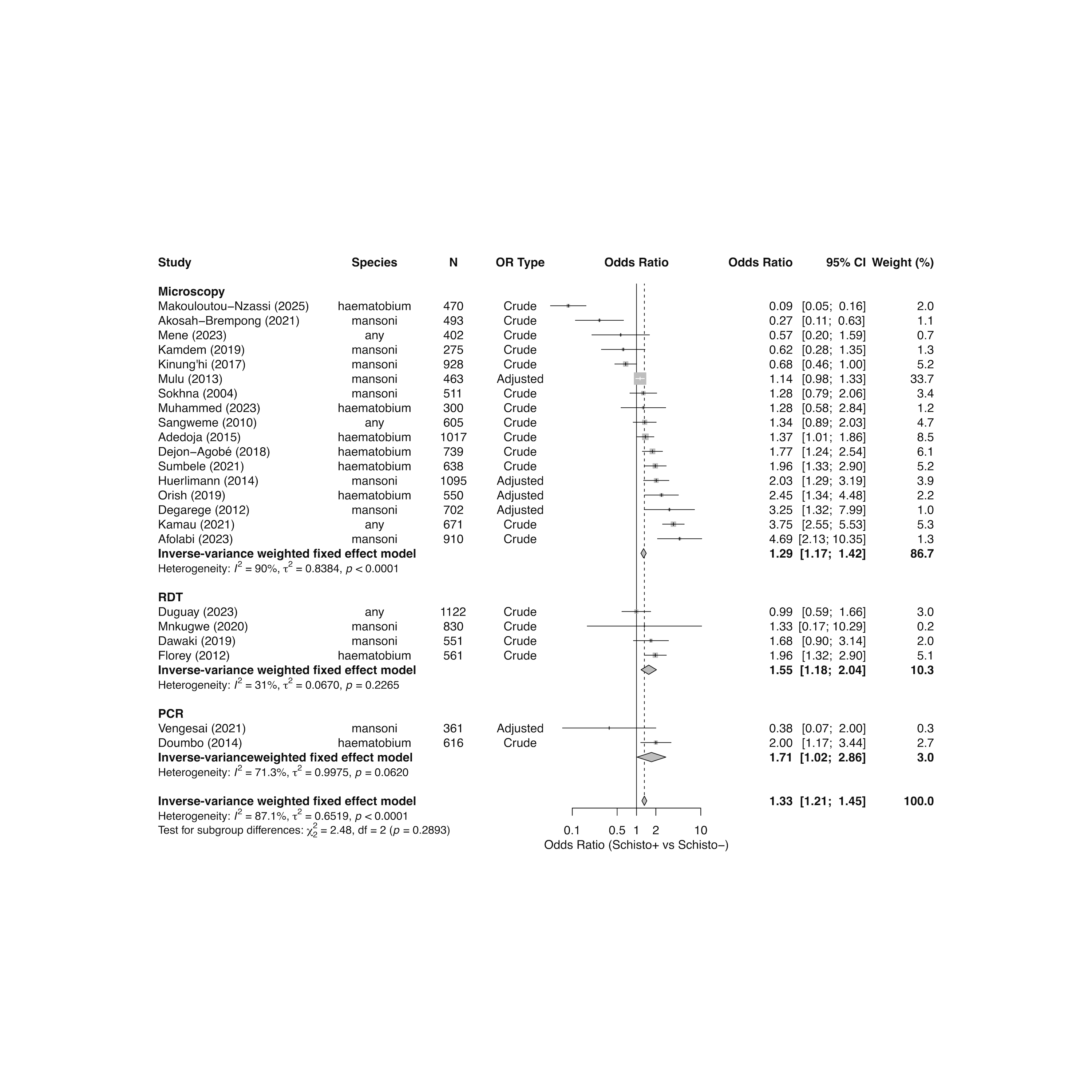

Supplement: S4 Fig — (TIFF) [file pntd.0014369.s004.tiff]

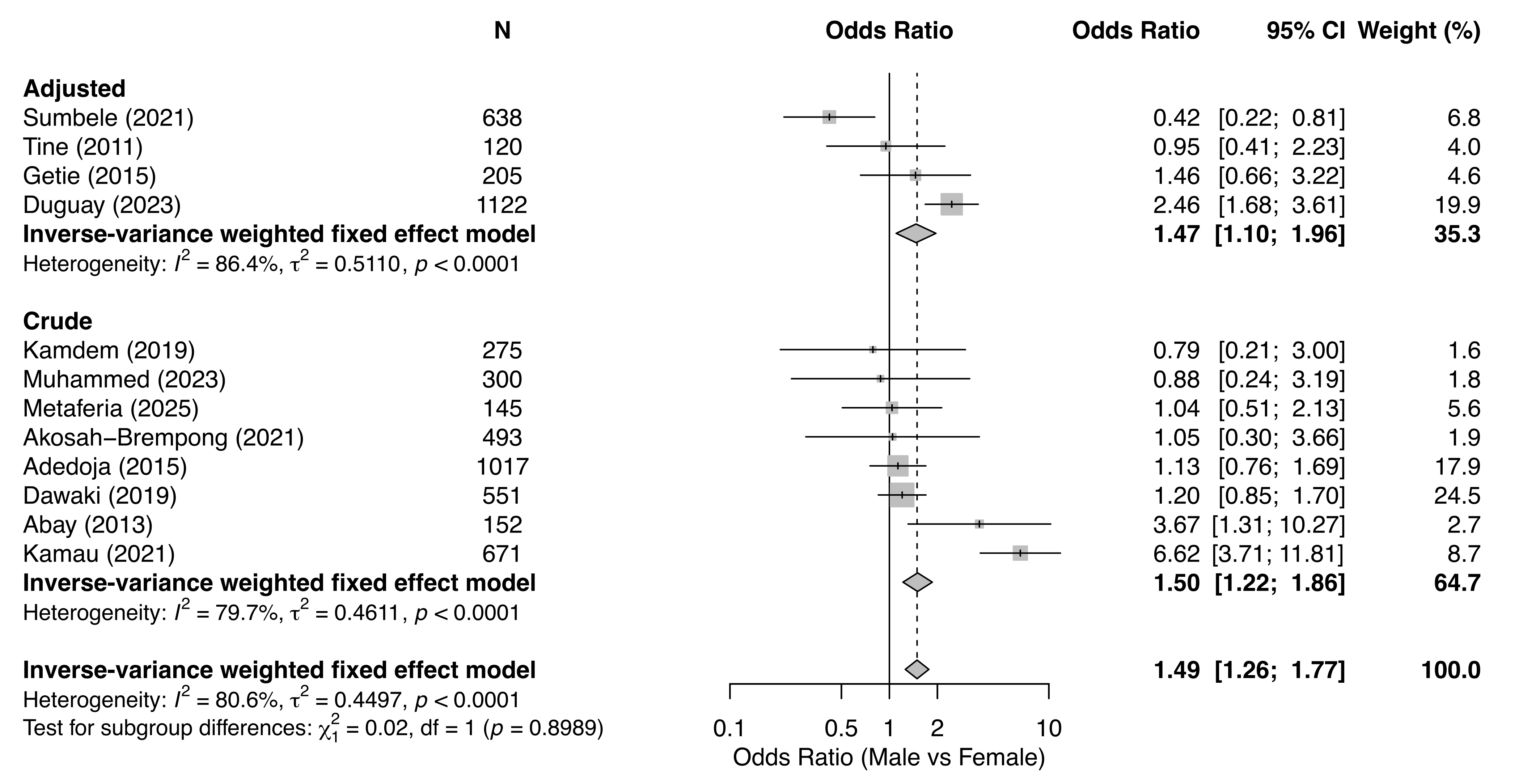

Supplement: S6 Fig — (TIFF) [file pntd.0014369.s006.tiff]

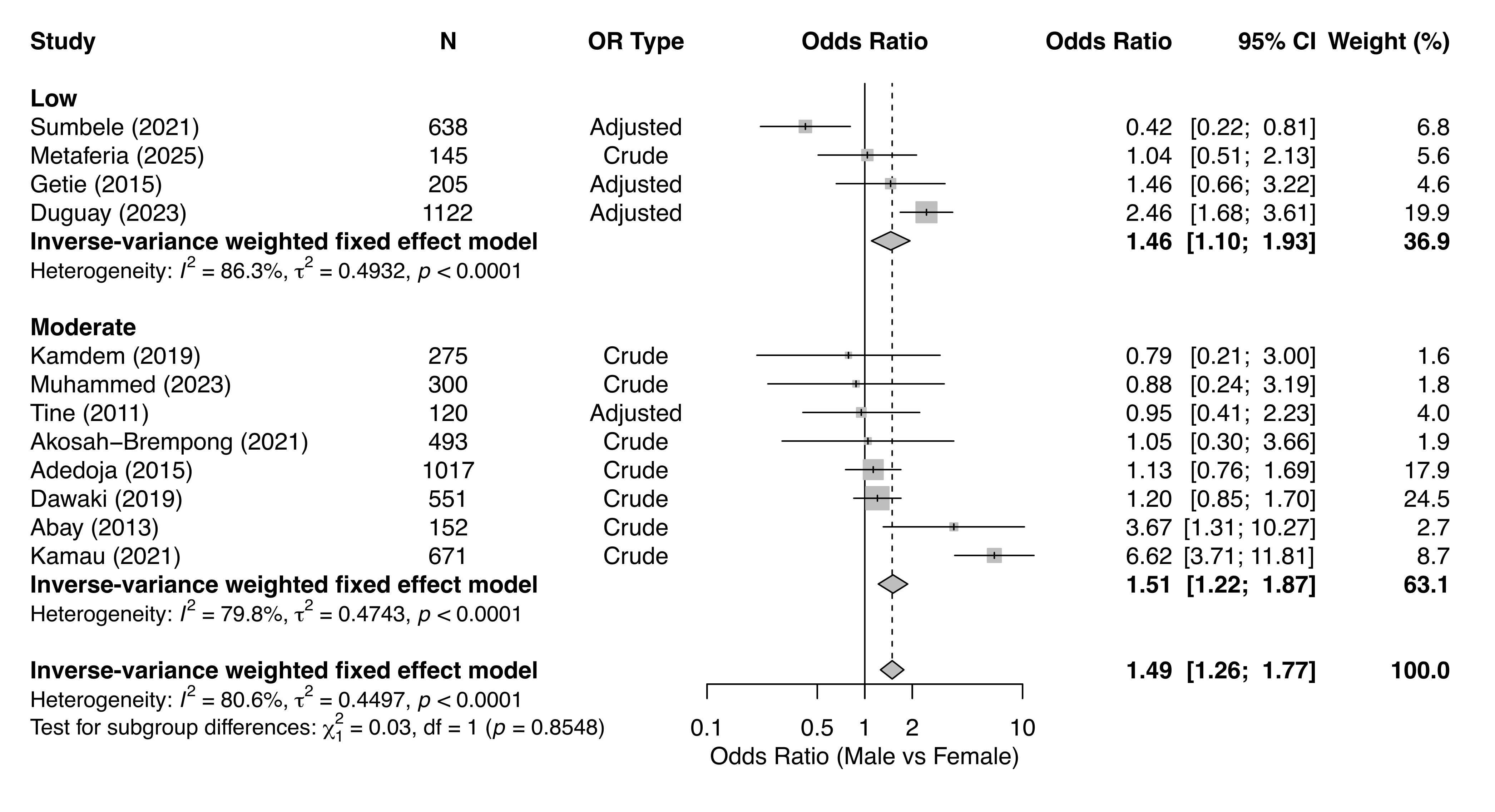

Supplement: S7 Fig — (TIFF) [file pntd.0014369.s007.tiff]
