# Supplementary material for: Shared risk factors for malaria and schistosomiasis co-infection: A systematic review and meta-analysis
Source: PLoS Negl Trop Dis. 2026 Jun 15;20(6):e0014369. doi: 10.1371/journal.pntd.0014369 (PMC13268186; doi:10.1371/journal.pntd.0014369)
Supplement: S3 Table — (DOCX) [file pntd.0014369.s010.docx]

**S3 Table. Summary of main findings and certainty of evidence (GRADE approach).** Certainty of evidence is based on GRADE principles for observational studies. By default, evidence from observational studies starts at ’Low’ certainty and an be downgraded for risk of bias, inconsistency (heterogeneity), indirectness, or imprecision.

| Risk Factor /  Exposure | No. of Studies | Pooled OR (95% CI) | Heterogeneity (*I*^2^) | Certainty of  Evidence | Primary Reasons for Downgrading |
| --- | --- | --- | --- | --- | --- |
| Schistosomiasis  infection | 23 | 1.27 (1.17–1.39) | 85.4% | Low ⊕⊕⊖⊖ | Observational designs; High unexplained  heterogeneity |
| Male sex | 13 | 1.49 (1.26–1.76) | 78.8% | Low ⊕⊕⊖⊖ | Observational designs;  High heterogeneity |
| Direct water contact | 3 | 2.53 (1.60–4.00) | 55.3% | Very Low ⊕⊖⊖⊖ | Observational designs;  Sparse data  (few studies);  Imprecise exposure  definitions |
