# Supplementary material for: Shared risk factors for malaria and schistosomiasis co-infection: A systematic review and meta-analysis
Source: PLoS Negl Trop Dis. 2026 Jun 15;20(6):e0014369. doi: 10.1371/journal.pntd.0014369 (PMC13268186; doi:10.1371/journal.pntd.0014369)
Supplement: S1 Text — (DOCX) [file pntd.0014369.s011.docx]

# Search Strategy

Logic of the search string is as follows (categories connected by ‘AND’):

| **Disease 1** | **Disease 2** | **Co-Infection** |
| --- | --- | --- |
| (malaria OR Plasmodium OR Plasmodium falciparum OR “P. falciparum” OR “Plasmodium vivax" OR malarial parasite) | (schistosom* OR schistosomiasis OR schistosoma OR schistosome OR bilharzia* OR Schistosoma mansoni OR Schistosoma haematobi* OR helminthiasis OR snail fever) | (co-infection* OR "dual infection" OR "co-occurrence" OR cooccur* OR “co-distributed” OR “co-distribution” OR codistribut* OR "concomitant" OR polyparasitism OR multiparasitism OR multi-parasitism OR multi-infection OR multiinfection OR “simultaneous infection” OR “sequential infection “OR joint infection OR superinfection OR synergistic infection) |

The generic search string was adapted to suit constraints and functionalities of different databases. Medline, Embase and Global Health were searched through Ovid (https://www.wolterskluwer.com/en/solutions/ovid). Web of Science was accessed through Clarivate (https://clarivate.com).

## OVID (703 Results)

### Medline 192

| ***#*** | **Query** | **Results from 19 Feb 2025** |
| --- | --- | --- |
| ***1*** | (malaria or Plasmodium or Plasmodium falciparum or "P. falciparum" or "Plasmodium vivax" or malarial parasite).mp. [mp=title, book title, abstract, original title, name of substance word, subject heading word, floating sub-heading word, keyword heading word, organism supplementary concept word, protocol supplementary concept word, rare disease supplementary concept word, unique identifier, synonyms, population supplementary concept word, anatomy supplementary concept word] | 125,708 |
| ***2*** | (schistosom* or schistosomiasis or schistosoma or schistosome or bilharzia* or Schistosoma mansoni or Schistosoma haematobi* or helminthiasis or snail fever).mp. [mp=title, book title, abstract, original title, name of substance word, subject heading word, floating sub-heading word, keyword heading word, organism supplementary concept word, protocol supplementary concept word, rare disease supplementary concept word, unique identifier, synonyms, population supplementary concept word, anatomy supplementary concept word] | 48,617 |
| 3 | (co-infection* or "dual infection" or "co-occurrence" or cooccur* or "co-distributed" or "co-distribution" or codistribut* or "concomitant" or polyparasitism or multiparasitism or multi-parasitism or multi-infection or multiinfection or "simultaneous infection" or "sequential infection OR joint infection" or superinfection or synergistic infection).mp. [mp=title, book title, abstract, original title, name of substance word, subject heading word, floating sub-heading word, keyword heading word, organism supplementary concept word, protocol supplementary concept word, rare disease supplementary concept word, unique identifier, synonyms, population supplementary concept word, anatomy supplementary concept word] | 275,213 |
| 4 | 1 and 2 and 3 | 192 |

### Embase 345

| ***#*** | **Query** | **Results from 19 Feb 2025** |
| --- | --- | --- |
| ***1*** | (malaria or Plasmodium or Plasmodium falciparum or "P. falciparum" or "Plasmodium vivax" or malarial parasite).mp. [mp=title, book title, abstract, original title, name of substance word, subject heading word, floating sub-heading word, keyword heading word, organism supplementary concept word, protocol supplementary concept word, rare disease supplementary concept word, unique identifier, synonyms, population supplementary concept word, anatomy supplementary concept word] | 156408 |
| ***2*** | (schistosom* or schistosomiasis or schistosoma or schistosome or bilharzia* or Schistosoma mansoni or Schistosoma haematobi* or helminthiasis or snail fever).mp. [mp=title, book title, abstract, original title, name of substance word, subject heading word, floating sub-heading word, keyword heading word, organism supplementary concept word, protocol supplementary concept word, rare disease supplementary concept word, unique identifier, synonyms, population supplementary concept word, anatomy supplementary concept word] | 53670 |
| 3 | (co-infection* or "dual infection" or "co-occurrence" or cooccur* or "co-distributed" or "co-distribution" or codistribut* or "concomitant" or polyparasitism or multiparasitism or multi-parasitism or multi-infection or multiinfection or "simultaneous infection" or "sequential infection OR joint infection" or superinfection or synergistic infection).mp. [mp=title, book title, abstract, original title, name of substance word, subject heading word, floating sub-heading word, keyword heading word, organism supplementary concept word, protocol supplementary concept word, rare disease supplementary concept word, unique identifier, synonyms, population supplementary concept word, anatomy supplementary concept word] | 396025 |
| 4 | 1 and 2 and 3 | 345 |

### Global Health 166

| ***#*** | **Query** | **Results from 19 Feb 2025** |
| --- | --- | --- |
| ***1*** | (malaria or Plasmodium or Plasmodium falciparum or "P. falciparum" or "Plasmodium vivax" or malarial parasite).mp. [mp=title, book title, abstract, original title, name of substance word, subject heading word, floating sub-heading word, keyword heading word, organism supplementary concept word, protocol supplementary concept word, rare disease supplementary concept word, unique identifier, synonyms, population supplementary concept word, anatomy supplementary concept word] | 106582 |
| ***2*** | (schistosom* or schistosomiasis or schistosoma or schistosome or bilharzia* or Schistosoma mansoni or Schistosoma haematobi* or helminthiasis or snail fever).mp. [mp=title, book title, abstract, original title, name of substance word, subject heading word, floating sub-heading word, keyword heading word, organism supplementary concept word, protocol supplementary concept word, rare disease supplementary concept word, unique identifier, synonyms, population supplementary concept word, anatomy supplementary concept word] | 39488 |
| 3 | (co-infection* or "dual infection" or "co-occurrence" or cooccur* or "co-distributed" or "co-distribution" or codistribut* or "concomitant" or polyparasitism or multiparasitism or multi-parasitism or multi-infection or multiinfection or "simultaneous infection" or "sequential infection OR joint infection" or superinfection or synergistic infection).mp. [mp=title, book title, abstract, original title, name of substance word, subject heading word, floating sub-heading word, keyword heading word, organism supplementary concept word, protocol supplementary concept word, rare disease supplementary concept word, unique identifier, synonyms, population supplementary concept word, anatomy supplementary concept word] | 43353 |
| 4 | 1 and 2 and 3 | 166 |

(malaria OR Plasmodium OR Plasmodium falciparum OR “P. falciparum” OR “Plasmodium vivax" OR malarial parasite)

AND

(schistosom* OR schistosomiasis OR schistosoma OR schistosome OR bilharzia* OR Schistosoma mansoni OR Schistosoma haematobi* OR helminthiasis OR snail fever)

AND

(co-infection* OR "dual infection" OR "co-occurrence" OR cooccur* OR “co-distributed” OR “co-distribution” OR codistribut* OR "concomitant" OR polyparasitism OR multiparasitism OR multi-parasitism OR multi-infection OR multiinfection OR “simultaneous infection” OR “sequential infection “OR joint infection OR superinfection OR synergistic infection)

## Web Of Science (307 Results)

(malaria OR Plasmodium OR Plasmodium falciparum OR “P. falciparum” OR “Plasmodium vivax" OR malarial parasite)

AND

(schistosom* OR schistosomiasis OR schistosoma OR schistosome OR bilharzia* OR Schistosoma mansoni OR Schistosoma haematobi* OR helminthiasis OR snail fever)

AND

(co-infection* OR "dual infection" OR "co-occurrence" OR cooccur* OR “co-distributed” OR “co-distribution” OR codistribut* OR "concomitant" OR polyparasitism OR multiparasitism OR multi-parasitism OR multi-infection OR multiinfection OR “simultaneous infection” OR “sequential infection “OR joint infection OR superinfection OR synergistic infection)

## Global Index Medicus (3 Results)

(malaria OR Plasmodium OR Plasmodium falciparum OR “P. falciparum” OR “Plasmodium vivax" OR malarial parasite)

AND

(schistosom* OR schistosomiasis OR schistosoma OR schistosome OR bilharzia* OR Schistosoma mansoni OR Schistosoma haematobi* OR helminthiasis OR snail fever)

AND

(co-infection* OR "dual infection" OR "co-occurrence" OR cooccur* OR “co-distributed” OR “co-distribution” OR codistribut* OR "concomitant" OR polyparasitism OR multiparasitism OR multi-parasitism OR multi-infection OR multiinfection OR “simultaneous infection” OR “sequential infection “OR joint infection OR superinfection OR synergistic infection)

## Pubmed (332 results)

(malaria OR Plasmodium OR Plasmodium falciparum OR “P. falciparum” OR “Plasmodium vivax" OR malarial parasite)

AND

(schistosom* OR schistosomiasis OR schistosoma OR schistosome OR bilharzia* OR Schistosoma mansoni OR Schistosoma haematobi* OR helminthiasis OR snail fever)

AND

(co-infection* OR "dual infection" OR "co-occurrence" OR cooccur* OR “co-distributed” OR “co-distribution” OR codistribut* OR "concomitant" OR polyparasitism OR multiparasitism OR multi-parasitism OR multi-infection OR multiinfection OR “simultaneous infection” OR “sequential infection “OR joint infection OR superinfection OR synergistic infection)
