# Supplementary material for: Shared risk factors for malaria and schistosomiasis co-infection: A systematic review and meta-analysis
Source: PLoS Negl Trop Dis. 2026 Jun 15;20(6):e0014369. doi: 10.1371/journal.pntd.0014369 (PMC13268186; doi:10.1371/journal.pntd.0014369)
Supplement: S2 Text — DOCX) [file pntd.0014369.s012.docx]

# Ratios

|  | M+ | M- | Total |
| --- | --- | --- | --- |
| S+ | **sex_n_male_co_infection_positive** | **sex_n_male_co_infection_negative** | schisto_n_positive |
| S- | **sex_n_male_co_infection_negative** | **sex_n_female_co_infection_negative** | schisto_n_negative |
| Total | Malaria_n_positive | Malaria_n_negative | **total_examined** |

## General

- In case of other diseases (triple infections etc.) they were also counted as co-infection
- If there was a co-infection of schisto species they were counted as co-infected S.mansoni + Malaria and in S.haematobieum + Malaria
- Need for clearer grouping and how one deals with polyparasitism
- Often not clear if groups are mutually exclusive or no
- cOR were calculated on Full Sample Characteristics
- Any column could also be triple infection

## Duguay 2023 [1]

## Sumbele 2021 [2]

- Also included Triple infection
- For cOR we considered triple infection just as co-infection so we would categorize individuals based on their status for only those two diseases, while still accounting for the entire study population.
- For aOR only reported the ones for “clean” co-infection

## Afolabi 2023 [3]

- Study was done cross sectionally but time points were in June and November 2021
- Prevalence was extracted from the 2x2 tables
- Numbers from Microscopy PCR and RDT and Combined do not line up with Gender Male and Female 🡪 nothing mentioned
- Gender in Table 3 is flipping around
- The "any Schistosomes" group should logically be composed of children with *S. haematobium*, children with *S. mansoni*, or children with both. If we assume the groups of 10 and 6 are not mutually exclusive (meaning some children have all three parasites), the total should be *less than or equal to* 16. So probably some children have all three parasites
- It is very confusing they mean P. falciparum + STH + S.
- haematobium + S. manson that basically “any helminth”

## Yapi 2014 [4]

- is not possible to provide the exact number of co-infections split by gender
- The study does not break down the 284 cases of *Schistosoma*-*Plasmodium* co-infection into counts of males and females.

## Tine 2011 [5]

- Not possible to extract 2x2 tables because only infection density and no absolute prealencec numbers

## Briand 2005 [6]

- were done in September, October, and December 2001 and January 2002. In addition, at recruitment in March 2002
- Because the study does not provide the raw numbers for how many of the 336 S. haematobium-positive children also had malaria, the table cannot be completed.
- The document lacks the specific number of coinfected individuals broken down by sex, which is required to complete the table.
- 2

## Kamau 2021 [7]

- Enrollment occurred between February 2017 and May 2018. The parent cohort study (RV393) was planned for a 24-month duration
- This was a sub-study using data and samples collected at the enrollment visit of a prospective observational cohort study (RV393)
- Fishing community in western Kenya
- The diagnostic test detects antigens for both species. The paper notes that *S. mansoni* is the most prevalent in the region , but the POC-CCA test has low sensitivity for *S. haematobium*
- Samples for both diagnoses were collected at the single enrollment visit.

## Mnkugwe 2020 [8]

- The study was conducted between February and May 2017.
- Analyses for malaria and haemoglobin were based on 824 children with complete data. The schistosomiasis prevalence was based on all 830 children.

## Abay 2013 [9]

- The study is described by the authors as a " (Pro study". Given that it follows a single group of co-infected patients before and after an intervention with no parallel control group, it is best described as a before-after study.
- The study was conducted in Kemise town. The study compared rural and urban residents but found no significant difference in co-infection rates.
- The study focused on uncomplicated *P. falciparum* malaria.

## Mulu 2013 [10]

- The study was conducted from November to December 2006.
- Inconsistency in Table 5 and full text used table 5 data

## Vengesai 2021 [11]

- Sample collection took place between February and May, though the specific year is not mentioned.
- ***Note on Data Inconsistency:*** The analyses for *S. mansoni* and *S. haematobium* were conducted on different subsamples of the total recruited population of 361 due to missing specimens. The tables below reflect the specific subsamples used for each analysis as reported in Table 1 of the

## Kamdem 2019 [12]

- Described as a cross-sectional study where samples were collected between Sept-Dec 2018. Stool samples were collected 5 days apart, but the exact timing of blood collection relative to stool collection is not stated.

## Kinung'hi 2017 [13]

- The study was cross-sectional and conducted in September 2013. This design does not involve a follow-up duration.
- BMC Research Notes
- As a cross-sectional study, all samples were collected during the same period in September 2013.
- The paper does not describe a statistical variable selection method (e.g., stepwise). Instead, it states the models were adjusted for a set of variables.

## Sokhna 2004 [14]

- The follow-up was conducted from September 1998 to April 1999.
- The study was in Richard Toll, located in the Senegal river basin near irrigated fields.
- Children were enrolled and followed prospectively to record malaria attacks.
- A further stool examination was performed in January 1999.
- Two slides were prepared from 25 mg of stool.

## Doumbo 2014 [15] / Doumbo 2018 [16]

- Prospective cohort study
- Clinical surveillance visits alternated between the study clinic and participants' homes.
- Baseline infection status for both pathogens was determined at enrollment. The outcome (febrile malaria) was monitored prospectively for 7 months.
- 2018 Another paper from 2018
- Tried to supplement the paper

## Degarege 2012 [17]

- This was a cross-sectional study. Data was collected over a 3-month period from December 2010 to February 2011.

- The study was conducted from December 2010 to February 2011.
- For calculating the Odds Ratios, "Individuals free from any intestinal helminth infection were used as reference categories".
- They ran check for people only with Schisto Mono infection but excluded the ones with tripled or quadruple infection 🡪 cant compare to other

## Muhammed 2023 [18]

- This was a cross-sectional study. Data was collected from June to August 2021.

## Lyke 2006 [19] / Lyke 2012 [20]

- Supplemented data but used initial study
- The study was conducted before the 2002 and 2003 malaria transmission seasons.
- Children were monitored weekly for 25 weeks during one malaria transmission season.
- The groups were matched by sex at enrollment, making this calculation irrelevant.

Adedoja 2015 [21] / Adedoja 2018 [22]

- Sublemented but second one was a genetic sub-study
- This was a cross-sectional study. Data was collected from October 2012 to May 2013.

Author Overlap Dr. Ayodele Adedoja is the lead author on both papers.

Location Both studies were conducted in Pategi, Kwara State, Nigeria.

Timing The previous study was conducted from October 2012 to May 2013. This study was conducted from March to May 2013, which is within the same time frame.

• Study Design The previous paper was a large cross-sectional prevalence study (n=1017). This paper is a smaller interventional sub-study (n=56 co-infected children) that likely recruited its participants from the larger screening effort

2018 explicitly cited

## Makouloutou-Nzassi 2025 [23]

Denominator for this is unclear (470 total or 212 Schisto-screened).

## Hürlimann 2019 [24]

The study pooled data from four community-based cross-sectional surveys and one national school-based cross-sectional survey.

The study also included hookworm as a primary helminth of interest.

Diagnosis of S. mansoni was by Kato-Katz microscopy. S. haematobium was diagnosed by urine filtration (microscopy) in community surveys and by reagent strips for microhematuria in the national school survey.

Pooled Analysis of Yapi 2014 [4] and Hürlimann 2014 [25]

## Hürlimann 2014 [25]

See above

## Sangweme 2010 [26]

- Parasitaemia was estimated by counting parasites against 200 white blood cells.
- Samples (blood, urine, stool) were collected concurrently at each time point (T0, T6, T12)

## Excluded because pooled analysis or other stuff available:

Hürlimann 2019 [24]

Yapi 2014 [4]

Adedoja 2015  [21]

Adedoja 2018 [22] (both sub-analysis of other 2015 paper)

Lyke 2012 [20] Substudy of Lyke 2006 [19]

Doumbo 2018 [16], Doumbo 2014 [15]

31 -1 with two models = 30 Studies

36

- 35 Adedoja 2015 [21]
- 34 Lyke 2012 [20]
- 33 Hürlimann 2019 [24]
- 32Yapi 2014 [4]
- 31 Adedoja 2018 [22]
- 30 Doumbo 2014 [15]

# Summary of Calculation Possibilities

This table outlines whether the 2x2 contingency tables could be calculated for each study based on the data provided in the source papers.

| **Study (Year)** | **Calculation Type** | **Possibility** | **Reason** |
| --- | --- | --- | --- |
| **Duguay (2023) [1]** | Sex vs. Co-infection | Not Possible | The breakdown of co-infection numbers by sex is not provided. |
|  | Schisto (Any) vs. Malaria | Possible | - |
| **Sumbele (2021) [2]** | Sex vs. Co-infection | Possible | - |
|  | Schisto (haematobium) vs. Malaria | Possible | - |
| **Orish (2019) [27]** | Sex vs. Co-infection | Not Possible | The sex of one child with a quadruple infection is not stated, preventing completion of the table. |
|  | Schisto (haematobium) vs. Malaria | Possible | - |
| **Afolabi (2023) [3]** | Sex vs. Co-infection | Possible | - |
|  | Schisto (All Species) vs. Malaria | Possible | Data could be inferred by subtracting subgroups from totals provided in the paper. |
| **Getie (2015) [28]** | Sex vs. Co-infection | Not Possible | Cannot be calculated because the underlying Schisto/Malaria table is not possible. |
|  | Schisto (mansoni) vs. Malaria | Not Possible | The entire study population was malaria-positive, so there is no malaria-negative comparison group. |
| **Metaferia (2025) [29]** | Sex vs. Co-infection | Not Possible | Cannot be calculated because the underlying Schisto/Malaria table is not possible. |
|  | Schisto (mansoni) vs. Malaria | Not Possible | The entire study population was malaria-positive, so there is no malaria-negative comparison group. |
| **Florey (2012) [30]** | Sex vs. Co-infection | Not Possible | The specific breakdown of how many co-infected individuals were male versus female is not provided. |
|  | Schisto (haematobium) vs. Malaria | Possible | - |
| **Adedoja (2015) [21]** | Sex vs. Co-infection | Possible | - |
|  | Schisto (haematobium) vs. Malaria | Possible | - |
|  | Schisto (mansoni) vs. Malaria | Not Possible | The specific number of children co-infected with both S. mansoni and P. falciparum is not provided. |
| **Oboh-Imafidon (2023) [31]** | Sex vs. Co-infection | Not Possible | The breakdown of co-infection numbers by sex is not provided. |
|  | Schisto (haematobium) vs. Malaria | Not Possible | The study population was composed entirely of S. haematobium-positive individuals, so there is no schisto-negative comparison group. |
| **Akosah‐Brempong (2021) [32]** | Sex vs. Co-infection | Possible | - |
|  | Schisto (All Species) vs. Malaria | Possible | - |
| **Yapi (2014) [4]** | Sex vs. Co-infection | Not Possible | The specific breakdown of co-infected children by sex is not provided. |
|  | Schisto (Any) vs. Malaria | Possible | - |
|  | Schisto (Species-specific) vs. Malaria | Not Possible | The paper does not provide specific co-infection numbers for S. mansoni and S. haematobium separately. |
| **Tine (2011) [5]** | Sex vs. Co-infection | Not Possible | The breakdown of the 55 co-infected children by sex is not provided. |
|  | Schisto (Any) vs. Malaria | Not Possible | The study was conducted exclusively among children who were already positive for malaria. |
| **Briand (2005) [6]** | Sex vs. Co-infection & Schisto vs. Malaria | Not Possible | The study lacks a single point-in-time prevalence for malaria to cross-tabulate with schistosomiasis data, making a standard 2x2 table impossible. |
| **Dawaki (2019) [33]** | Sex vs. Co-infection | Possible | - |
|  | Schisto (mansoni & haematobium) vs. Malaria | Possible | - |
|  | Schisto (Any) vs. Malaria | Not Possible | Combined schistosomiasis data also includes Soil-Transmitted Helminths and cannot be isolated. |
| **Dejon-Agobé (2018) [34]** | Sex vs. Co-infection | Not Possible | A breakdown of the co-infected group by sex is not specified. |
|  | Schisto (haematobium) vs. Malaria | Possible | - |
| **Kamau (2021) [7]** | Sex vs. Co-infection | Possible | - |
|  | Schisto (Any/mansoni) vs. Malaria | Possible | The CCA test used is primarily sensitive for S. mansoni, so "Any" and "mansoni" are treated as the same. |
| **Mnkugwe (2020) [8]** | Sex vs. Co-infection | Not Possible | The paper does not provide a breakdown of the co-infected group by sex. |
|  | Schisto (Any) vs. Malaria | Possible | - |
| **Abay (2013) [9]** | Sex vs. Co-infection | Not Possible | The underlying Schisto/Malaria table cannot be created as the entire study group was malaria-positive. |
|  | Schisto (mansoni) vs. Malaria | Not Possible | The entire study population was malaria-positive, meaning there was no negative control group for comparison. |
| **Mulu (2013) [10]** | Sex vs. Co-infection | Not Possible | The study does not provide a breakdown of co-infection cases by sex. |
|  | Schisto (mansoni) vs. Malaria | Possible | Based on explicit numbers from Table 5, despite a data inconsistency in the text. |
| **Vengesai (2021) [11]** | Sex vs. Co-infection | Not Possible | Data to populate the table is missing. |
|  | Schisto (mansoni & haematobium) vs. Malaria | Possible | - |
|  | Schisto (Any) vs. Malaria | Not Possible | The number of individuals co-infected with both S. mansoni and S. haematobium is not reported. |
| **Kamdem (2019) [12]** | Sex vs. Co-infection | Possible | - |
|  | Schisto (mansoni) vs. Malaria | Possible | - |
| **Kinung'hi (2017) [13]** | Sex vs. Co-infection | Not Possible | The specific number of co-infected boys or girls is not provided. |
|  | Schisto (mansoni) vs. Malaria | Possible | - |
| **Sokhna (2004) [14]** | Sex vs. Co-infection | Not Possible | The total number of male and female participants in the cohort is not stated. |
|  | Schisto (mansoni) vs. Malaria | Possible | - |
| **Doumbo (2014) [15]** | Sex vs. Co-infection | Not Possible | The study does not provide a breakdown of the 39 co-infected individuals by sex. |
|  | Schisto (haematobium) vs. Malaria | Possible | - |
| **Degarege (2012) [17]** | Sex vs. Co-infection | Not Possible | The study does not provide a breakdown of co-infected individuals by sex. |
|  | Schisto (mansoni) vs. Malaria | Possible | - |
| **Muhammed (2023) [18]** | Sex vs. Co-infection | Possible | - |
|  | Schisto (haematobium) vs. Malaria | Possible | - |
| **Lyke (2006) [19]** | Sex vs. Co-infection | Possible | - |
|  | Schisto vs. Malaria | Not Possible | The study has a prospective cohort design, not a cross-sectional one, making this calculation invalid. |
| **Makouloutou-Nzassi (2025) [23]** | Sex vs. Co-infection | Not Possible | The study does not provide a sex breakdown for the 31 co-infected children. |
|  | Schisto vs. Malaria | Not Possible | The malaria status of the schisto-negative children is not specified. |
| **Hürlimann (2019) [24]** | Sex vs. Co-infection | Not Possible | The paper does not provide a breakdown of the co-infected groups by sex. |
|  | Schisto (haematobium & mansoni) vs. Malaria | Possible | - |
| **Hürlimann (2014) [25]** | Sex vs. Co-infection | Not Possible | The paper does not break down the number of co-infections by sex. |
|  | Schisto (haematobium & mansoni) vs. Malaria | Possible | - |
| **Sangweme (2010) [26]** | Sex vs. Co-infection | Not Possible | The study does not report the number of co-infected children stratified by sex. |
|  | Schisto (Any) vs. Malaria | Possible | - |
| **Mene (2023) [35]** | Sex vs. Co-infection | Not Possible | The number of individuals co-infected with schistosomiasis and malaria is not stratified by sex. |
|  | Schisto (Any) vs. Malaria | Possible | - |

# References

1. Duguay C, Mosha JF, Lukole E, Mangalu D, Thickstun C, Mallya E, et al. Assessing risk factors for malaria and schistosomiasis among children in Misungwi, Tanzania, an area of co-endemicity: A mixed methods study. PLOS Glob Public Health. 2023;3(11):e0002468.

2. Sumbele IUN, Otia OV, Bopda OSM, Ebai CB, Kimbi HK, Nkuo-Akenji T. Polyparasitism with Schistosoma haematobium, Plasmodium and soil-transmitted helminths in school-aged children in Muyuka–Cameroon following implementation of control measures: a cross sectional study. Infect Dis Poverty. 2021;10(1):14.

3. Afolabi MO, Sow D, Mbaye I, Diouf MP, Loum MA, Fall EB, et al. Prevalence of malaria-helminth co-infections among children living in a setting of high coverage of standard interventions for malaria and helminths: two population-based studies in Senegal. Front Public Health. 2023;11:1087044.

4. Yapi RB, Hürlimann E, Houngbedji CA, Ndri PB, Silué KD, Soro G, et al. Infection and Co-infection with Helminths and Plasmodium among School Children in Côte d’Ivoire: Results from a National Cross-Sectional Survey. PLoS Negl Trop Dis. 2014;8(6):e2913.

5. Tine RC, Faye B, Ndiaye JL, Ndour CT, Brasseur P, Olliaro P, et al. Co-infection paludisme bilharziose urinaire chez les enfants d’âge scolaire en zone rurale sénégalaise. Med Mal Infect. 2011;41(2):112–4.

6. Briand V, Watier L, Hesran JYL, Garcia A, Cot M. Coinfection with Plasmodium falciparum and Schistosoma haematobium: protective effect of schistosomiasis on malaria in Senegalese children? Am J Trop Med Hyg. 2005;72(6):702–7.

7. Kamau E, Yates A, Maisiba R, Singoei V, Opot B, Adeny R, et al. Epidemiological and clinical implications of asymptomatic malaria and schistosomiasis co-infections in a rural community in western Kenya. BMC Infect Dis. 2021;21(1):937.

8. Mnkugwe RH, Minzi OS, Kinung’hi SM, Kamuhabwa AA, Aklillu E. Prevalence and correlates of intestinal schistosomiasis infection among school-aged children in North-Western Tanzania. PLoS One. 2020;15(2):e0228770.

9. Abay SM, Tilahun M, Fikrie N, Habtewold A. Plasmodium falciparum and Schistosoma mansoni coinfection and the side benefit of artemether-lumefantrine in malaria patients. J Infect Dev Ctries. 2013;7(6):468–74.

10. Mulu A, Legesse M, Erko B, Belyhun Y, Nugussie D, Shimelis T, et al. Epidemiological and clinical correlates of malaria-helminth co-infections in southern Ethiopia. Malar J. 2013;12:227.

11. Vengesai A, Kasambala M, Mutandadzi H, Mduluza-Jokonya TL, Mduluza T, Naicker T. Association of TNF (rs1800629) promoter polymorphism and schistosomiasis with sub-microscopic asymptomatic Plasmodium falciparum infections in a schistosomiasis-endemic area in Zimbabwe. Trop Med Int Health. 2021;26(3):366–373.

12. Kamdem SD, Konhawa F, Kuemkon EM, Meyo Kamguia L, Tchanana GK, Nche F, et al. Negative Association of Interleukin-33 Plasma Levels and Schistosomiasis Infection in a Site of Polyparasitism in Rural Cameroon. Front Immunol. 2019;10:2827.

13. Kinung’hi SM, Mazigo HD, Dunne DW, Kepha S, Kaatano G, Kishamawe C, et al. Coinfection of intestinal schistosomiasis and malaria and association with haemoglobin levels and nutritional status in school children in Mara region, Northwestern Tanzania: a cross-sectional exploratory study. BMC Res Notes. 2017;10(1):583.

14. Sokhna C, Le Hesran JY, Mbaye PA, Akiana J, Camara P, Diop M, et al. Increase of malaria attacks among children presenting concomitant infection by Schistosoma mansoni in Senegal. Malar J. 2004;3:43.

15. Doumbo S, Tran TM, Sangala J, Li S, Doumtabe D, Kone Y, et al. Co-infection of Long-Term Carriers of Plasmodium falciparum with Schistosoma haematobium Enhances Protection from Febrile Malaria: A Prospective Cohort Study in Mali. PLoS Negl Trop Dis. 2014;8(9):e3154.

16. Doumbo SN, Ongoïba A, Doumtabe D, Tran TM, Traoré A, Sangala J, et al. Prevalence of Malaria, Intestinal and Urinary parasite infections in Kalifabougou, Mali. Mali Med. 2018;33(1):10–5.

17. Degarege A, Legesse M, Medhin G, Animut A, Erko B. Malaria and related outcomes in patients with intestinal helminths: a cross-sectional study. BMC Infect Dis. 2012;12(1):291.

18. Muhammed H, Balogun JB, Dogara MM, Adewale B, Ibrahim AA, Okolugbo CB, et al. Co-infection of urogenital schistosomiasis and malaria and its association with anaemia and malnutrition amongst schoolchildren in Dutse, Nigeria. S Afr J Sci. 2023;119(7-8):1–2.

19. Lyke KE, Dabo A, Sangare L, Arama C, Daou M, Diarra I, et al. Effects of concomitant Schistosoma haematobium infection on the serum cytokine levels elicited by acute Plasmodium falciparum malaria infection in Malian children. Infect Immun. 2006;74(10):5718–24.

20. Lyke KE, Dabo A, Arama C, Daou M, Diarra I, Wang A, et al. Reduced T regulatory cell response during acute Plasmodium falciparum infection in Malian children co-infected with Schistosoma haematobium. PLoS One. 2012;7(2):e31647.

21. Adedoja A, Tijani BD, Akanbi AA, Ojurongbe TA, Adeyeba OA, Ojurongbe O. Co-endemicity of Plasmodium falciparum and Intestinal Helminths Infection in School Age Children in Rural Communities of Kwara State Nigeria. PLoS Negl Trop Dis. 2015;9(7):e0003940.

22. Adedoja A, Hoan NX, Van Tong H, Adukpo S, Tijani DB, Akanbi AA, Meyer CG, Ojurongbe O, Velavan TP. Differential contribution of interleukin‐10 promoter variants in malaria and schistosomiasis mono‐and co‐infections among Nigerian children. Trop Med Int Health. 2018;23(1):45-52.

23. Makouloutou-Nzassi P, Kouna LC, Mbani Mpega Ntigui CN, Longo-Pendy NM, Bourobou Bourobou JA, Bangueboussa F, et al. Asymptomatic Malaria Infection and Hidden Parasitic Burden in Gabonese Schoolchildren: Unveiling Silent Co-Infections in Rural and Urban Settings. Trop Med Infect Dis. 2024;10(1):11.

24. Hürlimann E, Houngbedji CA, Yapi RB, N’Dri PB, Silué KD, Ouattara M, et al. Antagonistic effects of Plasmodium-helminth co-infections on malaria pathology in different population groups in Côte d’Ivoire. PLoS Negl Trop Dis. 2019;13(1):e0007086.

25. Hürlimann E, Yüksel C, Houngbedji CA, Yéo B, Yapi RB, N’Dri PB, et al. Effect of deworming on school-aged children’s physical fitness, cognition and clinical parameters in a malaria-helminth co-endemic area of Côte d’Ivoire. BMC Infect Dis. 2014;14:411.

26. Sangweme DT, Midzi N, Zinyowera-Mutapuri S, Mduluza T, Diener-West M, Kumar N. Impact of schistosome infection on Plasmodium falciparum Malariometric indices and immune correlates in school age children in Burma Valley, Zimbabwe. PLoS Negl Trop Dis. 2010;4(11):e882.

27. Orish VN, Ofori-Amoah J, Amegan-Aho KH, Osei-Yeboah J, Lokpo SY, Osisiogu EU, et al. Prevalence of Polyparasitic Infection Among Primary School Children in the Volta Region of Ghana. Open Forum Infect Dis. 2019;6(4):ofz153.

28. Getie S, Wondimeneh Y, Getnet G, Workineh M, Worku L, Kassu A, et al. Prevalence and clinical correlates of Schistosoma mansoni co-infection among malaria infected patients, Northwest Ethiopia. BMC Res Notes. 2015;8(1):480.

29. Metaferia Y, Seid A, Fenta GM, Weldehanna DG, Adamu A, Gedefie A. Prevalence and Associated Factors of Schistosoma mansoni and Other Intestinal Helminthes Co-Infection Among Malaria Positive Patients in Malaria Endemic Areas of Northeast, Ethiopia: A Cross-Sectional Study. Health Sci Rep. 2025;8(2):e70410.

30. Florey LS, King CH, Van Dyke MK, Muchiri EM, Mungai PL, Zimmerman PA, et al. Partnering parasites: evidence of synergism between heavy Schistosoma haematobium and Plasmodium species infections in Kenyan children. PLoS Negl Trop Dis. 2012;6(7):e1723.

31. Oboh-Imafidon MA, Torbit SM, Jacob S, Schroeter MN, Tucker AR, Ojurongbe O, et al. Severity of Schistosoma haematobium co-infection with malaria in school-children is potentially modulated by host CD14 gene variants. BMC Res Notes. 2023;16(1):199.

32. Akosah-Brempong G, Attah SK, Hinne IA, Abdulai A, Addo-Osafo K, Appiah EL, et al. Infection of Plasmodium falciparum and helminths among school children in communities in Southern and Northern Ghana. BMC Infect Dis. 2021;21(1):1259.

33. Dawaki S, Al-Mekhlafi HM, Ithoi I. The burden and epidemiology of polyparasitism among rural communities in Kano State, Nigeria. Trans R Soc Trop Med Hyg. 2019;113(4):169–82.

34. Dejon-Agobé JC, Zinsou JF, Honkpehedji YJ, Ateba-Ngoa U, Edoa JR, Adegbite BR, et al. Schistosoma haematobium effects on Plasmodium falciparum infection modified by soil-transmitted helminths in school-age children living in rural areas of Gabon. PLoS Negl Trop Dis. 2018;12(8):e0006663.

35. Meñe GR, Mpina MG, Lopelo A, Nyakarungu EL, Bijeri JR, Elo AM, Ondo FA, Garcia GA, Phiri WP, Ali AM, Agobé JC. Effects of age, gender and soil-transmitted helminth infection on prevalence of Plasmodium infection among population living in Bata District, Equatorial Guinea. Trop Med Infect Dis. 2023;8(3):149.
