# Supplementary material for: Shared risk factors for malaria and schistosomiasis co-infection: A systematic review and meta-analysis
Source: PLoS Negl Trop Dis. 2026 Jun 15;20(6):e0014369. doi: 10.1371/journal.pntd.0014369 (PMC13268186; doi:10.1371/journal.pntd.0014369)
Supplement: S3 Text — (DOCX) [file pntd.0014369.s013.docx]

# **Quality appraisal tool**

We used an adjusted version of the Quality Assessment Tool for Observational Cohort and Cross-Sectional Studies for Schistosomiasis based on [1] and National Institutes of Health (NIH, Bethesda, MD, United States of America) to perform the quality appraisal [2]. The adjusted tool for Malaria and Schistosomiasis was applied to all eligible study designs (cohort studies, cross-sectional studies, case-control studies, before-after studies, randomised controlled trials).

## **Quality assessment categories**

| **Category** | **Question** | **Select 'yes' if** | **Select ‘no’ if** |
| --- | --- | --- | --- |
| Aim | Was the research question or objective in this paper clearly stated? | Authors clearly describe goal of their research and state the population, exposure and outcome, e.g. the aim was to identify risk factors of malaria and schistosomiasis co-infection in children | No aim or unspecific aim, e.g. this study examines the Malaria Schistosome co-infection patterns of an isolated farm-worker community |
| Representativeness | Was the study population clearly specified and defined? Were inclusion and exclusion criteria for being in the study prespecified and applied uniformly to all participants? (For case-control studies, were cases and controls clearly differentiated?) | Authors describe the group of people from which the study participants were selected or recruited, using demographics, location, and time period; e.g. school-age children, male and female (aged 5-14), enrolled on Oct 1, 2014, in the five schools in the catchment areas of study villages were eligible | Description lacking specifics on study population demographics, location, and time period, e.g. community members living in five communities were the study population |
| Representativeness | Is the sampling method clearly described? Is the sample representative of the population from which it is drawn and was a sample size justification, power description, or variance and effect estimates provided? (For RCTs was the method of randomisation described and is risk of bias low? For case-control studies, was selection of cases and controls clearly described?) | Sampling method clearly described, e.g. ten school-age children (5-14) per class in each of the five schools were sampled using stratified random sampling, statistical power was assessed | Lacking details to understand how sampling was done, e.g. 50 adults per village were selected for inclusion in the study, no justification for sample size provided |
| Representativeness | Response rate >50% or differences between respondents and non-respondents described? Was loss to follow-up after baseline 20% or less (if applicable)? | Provides statistics on response rate, e.g. 93% of respondents selected from village registries agreed to participate in the study, or differences in known characteristics of respondents and non-respondents (age, gender) were assessed using t-tests, loss to follow-up was 15% (if applicable) | Lacking information on response rate and differences between respondents and non-respondents provided |
| Exposure | Were risk factors for Malaria and Schistosome infection clearly defined and implemented consistently across all study participants? | **Yes:** Each shared risk factor (e.g., sociodemographic, environmental, spatial) has a clear definition provided in the study, and these definitions were applied consistently to all participants | No: Risk factor definitions are unclear, missing, or inconsistently applied across participants. |
| Outcomes | Were malaria and schistosomiasis diagnoses based on valid and reliable methods? | Diagnosis of malaria and schistosomiasis is confirmed through microscopy, antigen tests, or antibody tests. | The diagnostic methods are based on self-report, are not clearly described. |
| Outcomes | Was the definition of co-infection clearly stated and was the reference group clearly defined? | The study clearly defines co-infection, including whether it is concurrent or sequential and explicitly states the reference group. | The definition of co-infection and the resulting reference group is absent or ambiguous. |
| Outcomes | Were exposures and outcomes measured independently by two people? | Measurement and diagnostic test done by different people, e.g. self-reported water contact using survey (exposure) and microscopy by a trained technician (outcome) | Same person administering questions on water contact, mosquito exposure and infection, e.g. technicians responsible for microscopy (outcome measurement) recruited to contact direct water contact observation (exposure) |
| Analysis | Were adjusted effect estimates shown? For example, were age and gender adjusted for where relevant? | Studies report both types of models, e.g. unadjusted association between risk factors and infection status . Confidence Intervals are provided | Studies report only adjusted or unadjusted ORs/RRs, fail to provide 95% CIs |

**References**

1. Reitzug F, Ledien J, Chami GF. Associations of water contact frequency, duration, and activities with schistosome infection risk: A systematic review and meta-analysis. Freeman MC, editor. PLoS Negl Trop Dis. 2023;17: e0011377. doi:10.1371/journal.pntd.0011377

2. National Heart, Lung, and Blood Institute. Observational Cohort and Cross-Sectional Studies from the National Institutes of Health. Available: https://www.nhlbi.nih.gov/health-topics/study-quality-assessment-tools
