# Supplementary material for: Shared risk factors for malaria and schistosomiasis co-infection: A systematic review and meta-analysis
Source: PLoS Negl Trop Dis. 2026 Jun 15;20(6):e0014369. doi: 10.1371/journal.pntd.0014369 (PMC13268186; doi:10.1371/journal.pntd.0014369)
Supplement: S4 Text — (DOCX) [file pntd.0014369.s014.docx]

**S4 Text: Interpretation and assumptions for inverse-variance fixed effect(s) meta-analysis**

Following Rice et al., we distinguish the *estimator* used for pooling from the *model assumption* about how true study effects relate across studies. Let *β*^ˆ^*_i_* denote the estimate from study *i* (on a common scale, e.g. log(OR)), with standard error ˆ*σ_i_*. The inverse-variance pooled estimator is


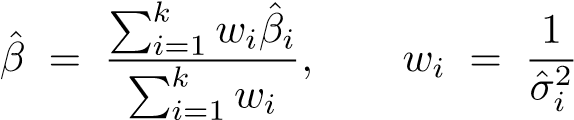
*.* (1)

Under standard large-sample conditions, its standard error is


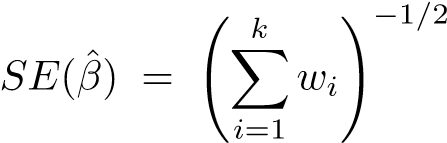
 *.* (2)

Rice et al. show that (1)–(2) remain statistically well-calibrated without assuming homogeneity of true effects. In particular, under a fixed effects (plural) model where each study has its own true effect *β_i_*, *β*^ˆ^ consistently estimates a precision-weighted average effect for the amalgamated population formed by combining the study populations:


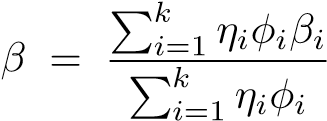
*,* (3)

where *η_i_* denotes the proportion of the amalgamated population contributed by study *i*, and *ϕ_i_* denotes the per-observation information accrual rate about *β_i_* (Fisher information rate). This interpretation differs from the *common effect model*, which assumes *β*_1_ = ··· = *β_k_*.

**Assumptions of inverse-variance fixed effect(s) inference**

1. The study estimates *β*^ˆ^*_i_* are independent across *i* (or approximately so).
2. Each *β*^ˆ^*_i_* is approximately normally distributed with variance *σ_i_*^2^, and ˆ*σ_i_* estimates *σ_i_* with error negligible relative to *σ_i_*.
3. Each *β*^ˆ^*_i_* targets a well-defined study-specific parameter *β_i_* on a common scale (e.g. consistent definition of exposure/outcome contrast and effect measure).
4. The pooled estimate *β*^ˆ^ is interpreted as a precisionweighted average across included study populations (Eq. 3), not as a single universal effect applicable to all settings.
5. Between-study variability is summarized (e.g. *I*^2^, *Q*) and explored using prespecified subgroup analyses / meta-regression rather than used as a gatekeeper for whether inversevariance pooling is “allowed”.

This Supplementary Text follows the framework and notation of Rice et al (2018).

**References**

[S1] Rice K, Higgins JP, Lumley T. A re-evaluation of fixed effect (s) meta-analysis. Journal of the Royal Statistical Society Series A: Statistics in Society. 2018 Jan;181(1):205-27.
