# Supplementary material for: Shared risk factors for malaria and schistosomiasis co-infection: A systematic review and meta-analysis
Source: PLoS Negl Trop Dis. 2026 Jun 15;20(6):e0014369. doi: 10.1371/journal.pntd.0014369 (PMC13268186; doi:10.1371/journal.pntd.0014369)
Supplement: Attachment — Submitted filename: Point-by-point-response.pdf [file pntd.0014369.s017.pdf]

# Reviewer Comments  Point by Point Response

## Reviewer #1

### Methods

The manuscript demonstrated a clear objective (i.e., evaluation of shared risk factors between schistosomiasis and malaria). The study also explicitly mentioned the kind of hypothesis it is going to address: (1) What are the risk factors associated with co-infection of malaria and schistosomes in humans? 2) What statistical assumptions have been made about the relationship between these infections in the literature?.

Systematic review and meta-analysis are the appropriate strategies to address the stated objective in the study.

The study population is clearly defined through a set of inclusion and exclusion criteria. The only drawback of such an approach is the restriction of the study to English literature, which could potentially introduce language bias. However, this is acknowledged by the authors as a limitation.
With the 30 studies included, out of which 27 were in the pooled analysis(16775 patients), the study has adequate power to test the hypothesis

The common effect model is reasonable as the studies had assessed similar outcomes. Moreover, appropriate subgroup analyses were done on the variables to account for the high heterogeneity.
There is no ethical concern

### Results

The analysis presented is congruent with the prespecified plan. Sub-group analyses and meta-analytical pooling were done appropriately. In addition, risk of bias assessment was thoroughly conducted via NIH criteria. Finally, narrative synthesis was included for age and socioeconomic status

The results are clearly presented with 95% CI and pooled effect sizes.

The figures and tables were well demonstrated and summarized in the text and were of good quality

The conclusion that the association between malaria and schistosomiasis is driven by environmental and behavioral exposures is well supported by the meta-analysis and narrative synthesis of the variables. However, it is essential to avoid the use of the word"driven by" as it may suggest causality which is unlikely given that most primary studies were observational The authors acknowledged the limitations of the analysis, mainly the reliance on English-language-based studies (potentially excluding local literatures), measurement variability across studies, residual high heterogeneity, inability to understand dynamic interactions, and scarcity of primary longitudinal studies.
It indicated that proper standardization of behavioural, environmental, and socioeconomic variables is crucial to enhance our understanding of co-infection in the future. In addition, more longitudinal research could shed light on the dynamic aspect of the interaction.
By recommending focused intervention on adolescents and young children, mostly males, in high-risk community settings, it addressed public health relevance

Overall, the systematic review and meta-analysis addressed highly relevant research topic in malaria and schistosomiasis endemic areas like Africa. It uses clear inclusion/exclusion criteria during selection, underwent thorough literature reviews, used sound methodology, and provided appropriate conclusions and recommendations. The main drawback of the study is the high residual heterogeneity that could impact precision.

**Author Response:**

We sincerely thank Reviewer #1 for their time, thorough evaluation, and feedback on our manuscript. We are encouraged that the reviewer found our objectives clear, our methodology sound, and our conclusions regarding shared environmental and behavioural exposures well-supported by the evidence. We appreciate the careful attention paid to our analysis and limitations.

Below, we have addressed the specific points raised in the review:

***Comment 1.1:*** *"The conclusion that the association between malaria and schistosomiasis is driven by environmental and behavioural exposures is well supported... However, it is essential to avoid the use of the word 'driven by' as it may suggest causality which is unlikely given that most primary studies were observational."*

**Author Response:** We thank the reviewer for their feedback on the causal language. We entirely agree that causal terms like "driven by" imply a level of causation that observational data cannot support. We have performed a thorough review of the manuscript and replaced causal terminology with associational language, such as "appears to be associated with" and "characterized by."

***Comment 1.2:*** *"The main drawback of the study is the high residual heterogeneity that could impact precision."*

**Author Response:** We appreciate the reviewer highlighting this point. We agree that the high residual heterogeneity ($I^2 > 80\%$) remains a key challenge in synthesizing this literature. As noted in our abstract and limitations section, while we explored this variance extensively

through subgroup analyses (e.g., by *Schistosoma* species, study setting, study design, and based on other reviewers suggestion based on diagnostic used), residual heterogeneity remained. To address the reviewer's concern, we have added a sentence to the limitations section explicitly outlining how this unexplained variance impacts the precision of our pooled estimates.

## Reviewer #2

The manuscript is a decent synthesis of shared risk variables for malaria–schistosomiasis co-infection. This publication tackles a significant public health concern in NTD epidemiology. The analysis is thorough, and the methodical approach is generally strict. After a major review, the material is timely, pertinent, and ready for publishing.

However, in order to improve validity, transparency, and policy relevance, certain methodological and interpretive flaws need to be fixed.

**Author Response:** We sincerely thank the reviewer for the assessment of our manuscript and for recognizing its relevance to public health and NTD epidemiology. We have carefully considered all comments and made the necessary revisions to enhance the validity, transparency, and policy relevance of our work. Below, we provide detailed point-by-point responses to each of the reviewer's comments.

### Minor Suggestions:

***Comment 1.1)*** *Explain why trials using various diagnostic techniques were combined without making any adjustments.*

**Author Response:** We thank the reviewer for raising this important methodological point. We explored the feasibility of stratifying our analyses by diagnostic technique.

For schistosomiasis, a meaningful subgroup analysis was not possible because most included studies relied on standard microscopy (Kato-Katz or urine filtration), with only one study utilizing an antibody test (sRDT) and one utilizing an antigen test (POC-CCA).

However, we were able to conduct a new subgroup meta-analysis for malaria diagnostics, grouping the studies into Microscopy (17 studies), RDT (4 studies), and PCR (2 studies). As demonstrated in the newly added Supplementary Figure S4, the test for subgroup differences was not statistically significant ($p = 0.2893$). While the point estimates varied (Microscopy OR 1.29; RDT OR 1.55; PCR OR 1.71), the overlapping confidence intervals and the formal

subgroup test indicate that the choice of malaria diagnostic did not significantly modify the pooled effect size, however hetereogenity in the RDT group was lower.

We have now added this forest plot to the supplementary materials and explicitly stated this rationale in both the Methods and Results sections.

***Comment 1.2)*** *Give age categories and the harmonization method more precise definitions.*

**Author Response:** We appreciate the reviewer's comment on the harmonization methodology for age categories. As the age brackets reported across the primary studies were highly heterogeneous and frequently overlapping (e.g., 6–14, 5–14, 8–17, and 16–20 years), quantitative harmonization (i.e., meta-analytical pooling) was not appropriate. As pre-specified in our registered protocol, we evaluated age using a narrative synthesis instead. We respectfully note that this rationale and our harmonization strategy are already explicitly defined in the final paragraph of the "Data analysis" section, which states:

*"For findings that could not be pooled (i.e., those reported by fewer than three studies or with different definitions, e.g., study sites, age groups), we performed a narrative synthesis by first summarizing the statistical methodologies..."*

Because this approach to unharmonizable variables is already clearly detailed in the manuscript, we have retained the current wording.

***Comment 1.3)*** *For readers who are not statisticians, provide a better explanation of the terms used in "common-effects models."*

**Author Response:** We agree with the reviewer that ensuring our statistical terminology is accessible to a broad public health audience is important. We have updated the "Data analysis" section to provide a clear, plain-language definition of the fixed-effects model and added a Supplementary Text S4 to ensure readers understand the underlying assumption being made. Our specific statistical justification for utilizing this model despite high heterogeneity is addressed in detail below in our response to Major Suggestion Comment 1.6).

***Comment 1.4)*** *Consider including a summary table of the main pooled effects with GRADE-style confidence and heterogeneity.*

**Author Response:** We thank the reviewer for this suggestion. We have added a sentence in the manuscript noting that all included studies were observational in design. Under GRADE criteria, the certainty of evidence would therefore be considered "Low" at baseline. A Summary of Findings table utilizing GRADE principles has been constructed for our three main quantitative pooled estimates (schistosomiasis infection, male sex, and water contact) and is now included as a supplementary table.

We have also expanded the Discussion to address this point, where we note that a key strength of observational studies in this context is their ability to explore a wide range of potential risk factors, thereby identifying priority areas for further experimental investigation. Identifying shared risk factors for co-infection can, by its nature, only be accomplished through observational or case-control designs. Once risk factors are established and targeted interventions are developed, future experimental studies (e.g., randomized controlled trials) would then be appropriate, at which point a formal GRADE assessment could yield higher-certainty evidence. We have also added a limitation statement acknowledging that, because the existing literature is entirely observational, caution is warranted when drawing direct policy recommendations from these findings.

## Major Suggestions:

***Comment 1.5) Risk of Bias Assessment Insufficiently Integrated,***

a) Discuss how methodological weaknesses (e.g., lack of clustering adjustment, unclear co-infection definitions, unmeasured confounding) may bias pooled estimates.

b) Add sensitivity analyses excluding moderate/high ROB studies.

**Author Response:** We thank the reviewer for highlighting the importance of robustly integrating the Risk of Bias (ROB) assessment.

Regarding point **(1.5b)**, we respectfully note that this analysis is already included in our Results section. We performed subgroup meta-analyses stratifying by ROB (Low, Moderate, and High) for our primary outcomes, which allows readers to see the isolated effects for each ROB group (see e.g., line 268 to 272, and 301 to 305).

Also, in the appendix for Schistosoma infection as a risk factor (Fig S5) for Malaria and sex as a risk factor (Fig S7), we estimated the pooled effect for each risk of bias group, which covers the requested sensitivity analysis.

Regarding point **(1.5a)**, we strongly agree that the specific methodological flaws of the primary literature must be connected to the potential bias of our pooled estimates. We appreciatively note that we already critique the lack of spatial clustering adjustment (in the *Statistical methodologies* section Line 327) and unmeasured confounding (in the *Discussion, Line 429 to 431*).

However, we thank the reviewer for the prompt to expand on the issue of "unclear co-infection definitions," as this is a systemic issue in the current literature. To our knowledge, while disease-specific definitions exist in various public health materials (such as WHO guidelines for Hepatitis B ("Coinfection occurs when a person is infected simultaneously with HBV and HDV[...]" or the NIH Glossary for HIV, which defines it as having *"two or more infections at the same time"*), there is no universal, standardized definition for co-infection, particularly within the context of neglected tropical diseases. Therefore, we oriented our framework around these established infectious disease definitions, broadly defining co-infection for our review as the *concurrent or sequential occurrence of both infections, regardless of temporal order.*

While primary studies often fail to distinguish between concurrent or sequential infections, we note that as most of our included studies (76.7%, 23/30) were cross-sectional, they inherently captured concurrent infections at a single time point. Therefore, while the lack of a standardized, field-wide definition is a limitation, its practical impact on introducing temporal misclassification bias into our specific pooled estimates is likely minimal. We have expanded our Limitations section to address this definitional gap, and we have added a call to action for the establishment of an explicit, standardized definition for NTD co-infections in future research.

***Comment 1.6)*** *Choice of Fixed-Effects Model Despite High Heterogeneity*

a) *The authors report very high heterogeneity in almost all pooled analyses ($I^2 > 80\%$).*
b) *Nevertheless, they apply a fixed-effects (common-effects) model throughout.*
c) *Re-run primary meta-analyses using random-effects models.*
d) *Present both models or justify clearly why fixed-effects was retained.*

**Author Response:** We thank the reviewer for raising this important point. We agree that heterogeneity is substantial in multiple pooled analyses (often $I^2 > 80\%$) and requires careful interpretation. We therefore appreciate this opportunity clarify the estimand and assumptions.

Our meta-analytic summary is the inverse-variance weighted estimator, sometimes referred to as fixed effect (or common-effect) meta-analysis. Let ($\widehat{\beta_i}$ ) denote the study-specific estimate

(e.g., log odds ratio) and ({^σ}_i) its standard error. We compute the pooled estimate and its standard error as follows

$$\hat{\beta};=;\frac{\sum_{i=1}^{k} w_i\widehat{\beta_i}}{\sum_{i=1}^{k} w_i}, \quad \text{where} \quad w_i = \frac{1}{\widehat{\sigma_i^2}}, SE(\hat{\beta}) = \sqrt{\frac{1}{\sum_{i=1}^{k} w_i}}$$

As emphasized by Rice et al. [1], use of $\hat{\beta}$ need not be interpreted as assuming a single common underlying effect across studies. Rice et al. distinguish between a common-effect model (($\beta_1 = \cdots = \beta_k = \beta$)) and a fixed effects model in which each study has its own underlying effect ($\beta_i$) without assuming exchangeability or a mixing distribution.

Under this relaxed fixed effect assumption, the inverse-variance pooled estimator ($\hat{\beta}$) is consistent for the precision-weighted average of the study-specific effects from the included study populations (Rice et al., Equation 3).

High $I^2$ does not invalidate inverse-variance pooling but indicates that the $\beta_i$ differ and thus $\beta$ should be interpreted as an average (with heterogeneity explored and reported). Rice et al. and the Cochrane Handbook for Systematic Reviews [2] explicitly argue that assessing the appropriateness of fixed effect meta-analysis by first checking homogeneity is not well-founded. Instead, the decision should be driven by whether the precision-weighted average effect is the scientific quantity of interest.

We did not adopt an inverse-variance random-effects model as the primary analysis because it targets a different estimand, typically the mean of an assumed mixing distribution of exchangeable effects, and relies on estimation of $\tau^2$ (between study variance), which is challenging for some of our meta-analyses (water contact) and sub-analysis with k<5 in certain groups. In addition, the random-effects weights

$w_i^{RE} = \frac{1}{\widehat{\sigma_i^2}+\widehat{\tau^2}}$ become similar when ($\widehat{\tau^2}$) is large relative to ($\widehat{\sigma_i^2}$), such that the pooled estimate approaches an approximately equal-weighted mean, thereby implicitly reweighting the underlying population.

To avoid terminological ambiguity, we updated all forest plots / figure captions that previously referred to a “common-effects model” to instead use “inverse-variance fixed effect model” (Rice et al., 2018 terminology), thereby avoiding any implication that we assumed a common (identical) underlying effect across studies.

We harmonized terminology throughout the Methods/Results/Discussion to use inverse-variance weighted fixed effect meta-analysis when referring to the estimator, and we avoid mixing *common-effect* and *fixed-effect* phrasing that can be interpreted as adopting the common-effect (homogeneity) assumption. In addition to the existing R code, we added a short Supplementary Text where we discuss assumptions explicitly and detail Rice et al.’s model distinction to clarify interpretation.

[1] Rice K, Higgins JP, Lumley T. A re-evaluation of fixed effect (s) meta-analysis. Journal of the Royal Statistical Society Series A: Statistics in Society. 2018 Jan;181(1):205-27.

[2] Cochrane Handbook for Systematic Reviews of Interventions. Chapter 10: Analysing data and undertaking meta-analyses. Cochrane. (Accessed 12 Mar 2026.)

## Original comments of the reviewer

### *Abstract*

***Comment 2.1)*** *Clarify the study objective and framework in the abstract (i.e., whether the focus is on co-infection, shared risk factors, or exposure–outcome relationships) to improve reader understanding of the scope of the review.*

**Author Response:** We appreciate the reviewer's attention to the clarity in the Abstract. We note that the primary focus of the review is explicitly stated in the title of the manuscript and the first sentence of the Abstract's Methods section (*"We conducted a systematic review and meta-analysis to identify shared risk factors for malaria-Schistosoma co-infection..."*.

***Comment 2.2)*** *Temper the interpretation of findings by avoiding causal language and briefly acknowledging the high heterogeneity across studies, which will strengthen the scientific accuracy of the abstract.*

**Author Response:** We fully agree with the reviewer on the importance of maintaining scientific accuracy. We have carefully reviewed the abstract and the entire manuscript to ensure all causal language (e.g., "driven by") has been replaced with appropriate associational terminology (e.g., "associated with"). We have explicitly acknowledged the variance in the Results section of the abstract, which clearly states: *"Heterogeneity was high (*$I^2$ *> 80\%).*

### *Introduction*

***Comment 2.3)*** *Make the conceptual framework clear at the outset, whether the emphasis is on interaction effects, shared exposure contexts, or actual biological co-infection.*

**Author Response:** We appreciate the reviewer's feedback. However, we note that our conceptual framework is explicitly established at the outset of the Introduction.

In the opening paragraph, we set the premise by stating: *"Individual co-infection with both Plasmodium and Schistosoma parasites is common... Despite the clinical importance, the risk factors for acquiring co-infections remain poorly understood."* We then immediately elaborate on this framework in the second paragraph by detailing the specific shared exposure contexts evaluated in the literature, such as sociodemographic variables and factors *"related to behavior and environment, such as proximity to water bodies or specific water contact*

*activities."* The emphasis is on shared risk factors for biological co-infection is already the central, stated focus of the opening paragraphs, we have retained the current wording.

***Comment 2.4)*** *By clearly outlining the shortcomings of earlier reviews and what this review specifically adds, you can strengthen the knowledge gap statement.*

**Author Response:** We agree with the reviewer that a clear knowledge gap statement is necessary. However, we note that the third and fourth paragraphs of the Introduction are already dedicated to outlining the limitations of prior reviews and defining the specific contribution of our study.

The text explicitly discusses how previous systematic reviews (such as Afolabi et al. and Ojo et al.) focused primarily on prevalence estimates and clinical outcomes in restricted populations or regions, rather than evaluating risk factors. We also summarize recent methodological and ecological meta-analyses (Powell-Romero et al., Duguay et al., Koellsch-Amet et al.) before concluding with the precise gap our study addresses: "To date, however, no review has systematically synthesized the individual-level risk factors associated with co-infection." As the shortcomings of the existing literature are already detailed in these paragraphs, we have maintained the current text.

***Comment 2.5)*** *Minimize the amount of background information about specific diseases and highlight how important their interactions are to public health.*

**Author Response:** We appreciate the reviewer's suggestion to keep the introduction concise. However, the manuscript currently provides only minimal background information on the specific diseases. The text restricts this context to just two sentences establishing global case numbers and the geographic overlap of at-risk populations. Providing this brief baseline is necessary to subsequently assess how distinct transmission pathways intersect to create co-infection risk.

Furthermore, the public health importance of their interaction is established immediately in the opening paragraph. The text explicitly highlights that dual infection is common and significantly impacts clinical outcomes before detailing the specific gaps in our understanding of shared risk factors. Because the introduction is already targeted and directly addresses the public health burden of co-infection, we have maintained the current wording.

***Comment 2.6)*** *Make the last paragraph better by clearly outlining the goals and hypotheses.*

**Author Response:** We appreciate the reviewer's comment regarding the clarity of our objectives. We respectfully note that the final paragraph of the Introduction already explicitly defines the study's goals through our defined research questions. Consequently, we have retained the current structure of the final paragraph.

*Materials and Methods*

***Comment 2.7)*** *Justify the use of a fixed-effects model or do a fresh analysis using random-effects models where there is substantial variability.*

**Author Response:** We thank the reviewer for giving us the opportunity to clarify this methodological decision. We refer to our explanation in the **Major Suggestions Comment 1.6).**

***Comment 2.8)*** *Describe co-infection precisely and set it apart from studies that treat one illness as an exposure and the other as a consequence.*

**Author Response:** We thank the reviewer for raising this point. As noted in our previous response regarding disease definitions (Comment 1.5), there is to our knowledge no standardized definition for co-infection in the context of neglected tropical diseases. To capture the available literature, we provided a specific working definition in the Methods ("Outcome and exposures"), describing it as the concurrent or sequential occurrence of both infections. Furthermore, in accordance with our registered protocol, we calculated crude odds ratios directly from 2x2 tables when studies provided sufficient raw data. This step allowed us to extract standardized effect sizes regardless of how the primary authors parameterized their models.

We explicitly separate and analyze these varying modeling strategies in our Results section under "Statistical methodologies and model setups.", where we detail how the predominant strategy treated co-infection as a distinct binary outcome, whereas other studies modeled one infection as an independent predictor for the other.

Importantly, we dedicate a substantial portion of our Limitations section to critiquing the flaws inherent in these approaches. We explicitly state: *"Further, this review is limited by the reliance on standard logistic regression to model co-infection as a simple binary outcome (co-infected vs. not co-infected). By collapsing the four distinct infection states (uninfected, malaria-only, schistosomiasis-only, and co-infected) into a dichotomy, these models fail to simultaneously estimate the probabilities of each parasite and the correlation between them. This leads to ambiguity regarding the reference population; comparing 'co-infected' individuals to a 'not co-infected' group compares uninfected individuals with those harboring single infections, potentially biasing risk estimates if the single-infection groups have divergent risk profiles."*

Because this distinction and the accompanying methodological critique are already thoroughly mapped out in both the Results and Discussion sections, we have maintained the existing text.

***Comment 2.9)*** *Provide more details on the handling of cluster-adjusted estimates and confounding variables.*

**Author Response:** We appreciate the reviewer's request for additional detail. We note that the extraction and handling of these variables is already explicitly detailed in our Results section, specifically under the heading "Statistical methodologies and model setups."

In that section, we report that only three of the thirty included studies (10.0%) accounted for spatial clustering using mixed-effects models or generalized estimating equations (GEE). We also outline that the adjustment for confounding factors was highly variable and predominantly relied on stepwise selection processes employing Likelihood Ratio Tests. The primary literature did not generate cluster-adjusted estimates. We prioritized adjusted odds ratios when available, as stated in our Methods (and pre-registered protocol), but were constrained by the underlying study designs and limited studies available.

To make the impact of this limitation clearer to the reader, we have added a brief sentence to our Discussion section reiterating how this variability in primary study design affects our synthesis.

***Comment 2.10)*** *Explain the rationale behind integrating studies utilizing many diagnostic methods.*

**Author Response:**

We thank the reviewer for raising this question. Because this point directly overlaps with Comment 1.1 regarding the combination of various diagnostic techniques without statistical adjustments, we direct the reviewer to our detailed response to Comment 1.1 above.

***Comment 2.11)*** *Explain the evaluation of bias risk and its impact on interpretation.*

**Author Response:**

We appreciate the reviewer raising this point. We provided a detailed response to Comment 1.5 regarding the integration of our risk of bias assessment and its effect on the pooled estimates and therefore direct the reviewer to our comprehensive response to Comment 1.5 above.

*Results*

***Comment 2.12)*** *Rearrange the findings to show the conceptual difference between exposure-outcome relationships and shared risk variables.*

**Author Response:** We appreciate the reviewer's suggestion regarding the organization of our findings. We note that the current manuscript structure already addresses this conceptual division. The results intentionally separate the analysis of *Schistosoma* infection as a discrete risk factor for malaria from the subsequent evaluation of shared sociodemographic and environmental variables for co-infection.

Because the primary literature overwhelmingly collapses co-infection into a simple binary outcome, these estimates effectively serve as a proxy for the residual correlation between the two diseases. From a statistical perspective, this relationship would be much more appropriately evaluated by modeling the joint distribution of both infections, a methodological limitation we discuss extensively in the manuscript.

Given that this analytical progression is already established and considering that other reviewers found the current structural outline clear, we have elected to maintain the original sequence of the text.

***Comment 2.13)*** *Provide important pooled estimates along with heterogeneity and confidence levels in a succinct summary table.*

**Author Response:** We appreciate the reviewer's suggestion to include a summary table of our main findings. Because this request directly aligns with the recommendation raised in Comment 1.4, we refer to our response above.

***Comment 2.14)*** *Analyses with limited included studies (such as water contact) should be interpreted with caution.*

**Author Response:** We agree that estimates derived from a small number of studies require cautious interpretation. We explicitly address this limitation in our Discussion section regarding the water contact analysis.

The manuscript details that the quantitative synthesis for this variable was restricted to only three studies. Furthermore, we dedicate a paragraph to outlining how the scarcity of data, the

reliance on unstandardized environmental proxies, and the inherent flaws of univariate modeling complicate the broader interpretation of this risk factor. The text specifically warns that standard modeling techniques cannot determine if water contact is a true driver of dual susceptibility or simply a statistical artifact driven by schistosomiasis alone. Because this necessary caution is already thoroughly integrated into our discussion of the findings, we have maintained the current text.

***Comment 2.15)*** *Any sensitivity analysis results should be clearly reported, or their lack should be explained.*

**Author Response:** We agree that detailing these analytical decisions is necessary for transparency. We respectfully direct the reviewer to the "Subgroup analysis" subsection of the Methods, where the rationale for both conducting and omitting specific stratifications is already explicitly stated.

The text outlines the specific subgroup and sensitivity analyses performed to explore heterogeneity, which include stratifications by *Schistosoma* species, study design, risk of bias, reference population, effect estimate type, study setting, and based on the reviewers comments now the malaria diagnostic method. Furthermore, the manuscript directly explains the absence of other stratifications. We specify that water contact was analyzed without stratification because only three studies provided extractable data. We also explain that a subgroup analysis for schistosomiasis diagnostics was not performed because all but two included studies relied exclusively on microscopy-based methods.

Because these parameters and their justifications are already clearly documented in the manuscript, we have maintained the existing text.

*Conclusions*

***Comment 2.16*** *Give a succinct summary of the most reliable results.* ***and Comment 2.17*** *Because of the significant variability in observational designs, avoid making too many generalizations.*

**Author Response:** We appreciate the reviewer’s guidance on framing our final remarks. The current conclusion provides a summary of our most reliable findings by identifying age and sex as well-established determinants, and it translates these findings into specific targeted groups (older children, adolescents, and males in high-risk settings). Furthermore, the text deliberately restricts generalizations by explicitly acknowledging the methodological

limitations of the primary literature. We note a "scarcity of standardized evidence" for behavioral and environmental dimensions and critique the field's heavy reliance on cross-sectional studies and univariate models.

***Comment 2.18*** *Clearly state the research priorities, particularly for interventional and longitudinal studies.* and

***Comment 2.19*** *Stress the importance of comprehensive NTD control methods.*

**Author Response:**

We thank the reviewer for highlighting these priorities. The necessity of comprehensive control methods is already a central focus of the conclusion. We explicitly stress this by calling for a transition *"from separate vertical programs to the integrated interventions envisioned in the WHO Neglected Tropical Disease Roadmap 2030."* We also clearly outline the need for longitudinal designs and standardized environmental metrics. To fully accommodate the reviewer's recommendation regarding research priorities, we have slightly modified the text to explicitly include a call for interventional studies.

*Discussion*

***Comment 2.19*** *When assessing cross-sectional data, steer clear of causative terminology and instead focus on relationships rather than causation.*

**Author Response:** We agree that cross-sectional data cannot support causal inferences. As mentioned in our response to Comment 1.2 and the general comments regarding the abstract, we have conducted a thorough review of the manuscript to ensure that causative terminology (e.g., "driven by," "impact of") has been replaced.

***Comment 2.20*** *Incorporate risk of bias findings more directly into the evidence certainty debate.*

**Author Response:** We agree that risk of bias is fundamental to the debate over evidence certainty. We respectfully note that we have already integrated these findings throughout the Results and Discussion. Specifically, we performed subgroup analyses for our primary outcomes (schistosome infection and sex) stratified by risk of bias, allowing for a direct comparison of effects between low-risk and moderate-to-high-risk studies.

In the Discussion, we also critique how methodological weaknesses identified during our assessment, such as the lack of spatial clustering adjustment, limit the precision and certainty

of the evidence. Consequently, we believe these findings are already thoroughly integrated into the manuscript.

***Comment 2.21*** *Extend the conversation about the ecological context and biological plausibility of the relationship between schistosomiasis and malaria.*

**Author Response:** We appreciate the suggestion to discuss these contexts. We believe the manuscript already addresses these dimensions in detail within the Discussion section.

Regarding the ecological context, we dedicate two paragraphs to analyzing shared exposure pathways. We specifically evaluate the role of direct water contact and environmental proxies (such as proximity to rivers or dams) and propose the adoption of standardized spatial metrics like NDVI and NDWI to better characterize these transmission environments.

Regarding biological plausibility, we include a paragraph evaluating the interaction between pathogens. Although studies focusing on continuous clinical outcomes were excluded from our meta-analysis, we discuss their findings to provide biological context, specifically referencing evidence of altered *P. falciparum* densities (Briand et al.), changes in clinical malaria episodes (Lyke et al.), and the role of host genetic factors in disease severity (Oboh-Imafidon et al.).

Because these ecological and biological discussions are already integrated into our characterization of co-infection risk, we have maintained the current text.

***Comment 2.22*** *Incorporate integrated surveillance, school-based interventions, and WASH methods into the section on policy implications.*

**Author Response:**

We thank the reviewer for this suggestion. We have added a short paragraph in the Discussion highlighting the potential role of integrated surveillance and interventions in addressing shared risk factors for malaria–schistosomiasis co-infection, while noting that the current evidence base remains limited.

## *References*

***Comment 2.23*** *Several references have uneven formatting (such as different journal name abbreviations, punctuation, and italics usage); the PLOS reference style should be followed throughout the reference list.*

**Author Response:**

We have revised the entire reference list to ensure consistent formatting in accordance with the PLOS reference style, including standardized journal name abbreviations, punctuation, and italics usage.

***Comment 3.24*** *Certain citations in the text should be carefully cross-checked for accuracy because they do not exactly match the reference list in terms of author order, year, or spelling.*

**Author Response:**

We have carefully cross-checked all in-text citations against the reference list and corrected any discrepancies in author order, year, or spelling.

***Comment 3.25*** *A few references seem to lack comprehensive bibliographic information, such as page numbers, volume, or DOI. Whenever possible, these should be finished.*

**Author Response:**

We have reviewed all references and added missing bibliographic details (e.g., page numbers, volume numbers, and DOIs) where available.

***Comment 3.26*** *Verify that the reference list contains all of the references listed in the text and that there are no uncited references left.*

**Author Response:**

We have verified that all in-text citations are present in the reference list and confirmed that no uncited references remain.

## *Grammatical Suggestions:*

| | Original Text | Issue | Suggested Correction |
|---|---|---|---|
| 41 | literature?. | Incorrect punctuation | literature? |

| | | | |
|---|---|---|---|
| 45 | CRD42025064882) | Inconsistent ID format | Should match the earlier mentioned in the abstract ID CRD420250648824 |
| 52 | ”P. falciparum” | Smart quotes inconsistent | Use standard quotes: " P. falciparum" |
| 69 | diarrhoea | Inconsistent spelling (British vs American) | Consider using consistent spelling throughout (e.g., diarrhea) |
| 85–86 | A randomly selected 10% of full-text publications | Awkward phrasing | Ten percent of full-text publications were randomly selected |
| 96 | schistosome-malaria | Inconsistent hyphenation | Use consistently: schistosome–malaria |
| 145 | I2-statistic | Formatting | $I^2$ statistic |
| 169 | Study characteristics (no punctuation) | Heading style inconsistency | Add a colon or formatting consistent with other headings |
| 200 | lack of an independent or blinded outcome | Incomplete phrase | lack of independent or blinded outcome assessment |
| 248 | type of effect measure | Slightly ambiguous | type of effect estimate |
| 297 | model Setups | Capitalization error | model setups |
| 305 | (Y ) | Extra space | (Y) |

| | | | |
|---|---|---|---|
| 330 | however | Missing comma | however, |
| 356 | Our findings reveal that the risk profile... | Long sentence | Consider splitting for clarity |
| 418–421 | Because water contact is... | Sentence fragment | Merge with the previous sentence |
| 456–457 | co-infected vs. not co-infected | Informal style | co-infected versus non–non-co-infected |

**Author Response:**

We thank the reviewer for these detail suggestions. We have adjusted the manuscript accordingly.

## Reviewer #3

***Comment 3)*** *the study is well conducted but these comments to enhance it:*
**Author Response:** We thank Reviewer #3 for their assessment of our study and for providing constructive feedback to further refine the manuscript. Below, we address each specific comment in detail.

### *Methods*

***Comment 3.1)*** *Should include clear definition of coinfection.*
**Author Response:** We agree that establishing a clear definition is a fundamental requirement. To our knowledge, while disease-specific definitions exist in various public health materials (such as WHO guidelines for Hepatitis B or the NIH Glossary for HIV), there is no universal, standardized definition for co-infection within the context of neglected tropical diseases. To capture the available literature, we provided a admittingly broad working definition in the Methods ("Outcome and exposures" section), describing it as the concurrent or sequential occurrence of both infections, as we wanted to include a wide range of studies.
Furthermore, we have expanded our Limitations section to explicitly discuss this field-wide definitional ambiguity. We note that because most of our included studies are cross-sectional, they inherently capture concurrent infections at a single time point, minimizing temporal misclassification bias. We have also added a call to action for the establishment of an explicit, standardized definition for NTD co-infections in future research.

*Results*

***Comment 3.2)*** *soften causal language as most of the studies used are cross sectional studies.*
**Author Response:** We fully agree with this assessment by the reviewer. Because the majority of the included primary literature utilizes cross-sectional designs, inferring causality is inappropriate. We have conducted a comprehensive review of the entire manuscript, including the Abstract, Results, Discussion, and Conclusion, to ensure that causative terminology (e.g., "driven by," "impact of") has been removed. We replaced these terms with accurate associational language (e.g., "associated with," "characterized by") to properly reflect the observational nature of the underlying data.

***Comment 3.3)*** add diagnostic variability in the limitations as it may affect the definition of coinfection.

**Author Response:** We appreciate this constructive suggestion. Diagnostic variability directly affects the sensitivity of detection and, consequently, the practical definition of co-infection within a given study. We have updated the Limitations section to explicitly discuss how the heavy reliance on standard microscopy, as opposed to more sensitive molecular techniques (like PCR), likely leads to the underestimation of light infections and introduces measurement bias. Additionally, to assess this impact, we performed a new subgroup meta-analysis stratifying by malaria diagnostic type (Microscopy vs. RDT vs. PCR). As demonstrated in the newly added Supplementary Figure S5, the test for subgroup differences was not statistically significant ($p = 0.2893$). We have added this forest plot to the supplementary materials and explicitly stated this rationale in both the Methods and Results sections.

***Comment 3.4)*** *introduction should highlight the risk of coinfection and how this review help in preventing coinfection.*

**Author Response:** We thank the reviewer for highlighting the translational public health value of this work. The opening paragraph of the Introduction establishes the risk profile by detailing the geographic overlap of at-risk populations and the severe clinical consequences of dual infection.

To better link these baseline risks to concrete prevention strategies, we have added a new paragraph to the Discussion section outlining specific policy implications. This new text translates our epidemiological findings into potential intervention strategies, however we note that the current evidence in the literature is quite limited.

***Comment 3.5)*** *Complete PRISMA checklist.*

To our knowledge, a complete PRISMA checklist was provided during the initial submission. However, we have thoroughly reviewed and updated the checklist for this resubmission to ensure that all items are accounted for and that the page numbers accurately reflect the revised manuscript.
