# Supplementary material for: Shared risk factors for malaria and schistosomiasis co-infection: A systematic review and meta-analysis
Source: PLoS Negl Trop Dis. 2026 Jun 15;20(6):e0014369. doi: 10.1371/journal.pntd.0014369 (PMC13268186; doi:10.1371/journal.pntd.0014369)
Supplement: Attachment — Submitted filename: Point-by-point-response_auresp_2.pdf [file pntd.0014369.s019.pdf]

Reviewer Comments Point by Point Response

General Comments:

The clarity, organization, and methodological transparency of the revised manuscript have been significantly improved. Several major issues from the first round have been addressed by the writers, especially about:

1) More precise expression of goals.

2) Better methodological explanation (subgroup analyses, search approach).

3) Extended conversation about interpretation and heterogeneity.

4) Improved correlation between results and public health significance.

Response:
We sincerely thank the reviewer for their thorough and constructive evaluation of our manuscript, as well as acknowledging the improvements made. We are pleased that these revisions were well-received. Below, we address the remaining comments point by point.

Reviewer 2 Comments

Abstract

Comment 1.1 Please consider changing the language that is causal:"more frequently co-occurring" to "associated with higher odds".

Response: We thank the reviewer for this comment. The exact phrase "more frequently co-occurring" does not appear in our current Abstract. We assume the comment may be paraphrasing the opening of the Background section ("resulting in frequent co-infection"). This could appear to be emphasising how often two infections appear together, rather than the associational (odds-based) evidence our review summarizes. We have therefore changed that wording, using the language recommended.

Comment 1.2 Include a statement that acknowledges: High heterogeneity and how it affects interpretation.

Response: We thank the reviewer for emphasising the importance of the high heterogeneity we observed in our analysis. We respectfully note that the current abstract already states high

heterogeneity in a standalone sentence, immediately after the pooled effect sizes. We extended this sentence to warrant caution with respect to interpretation.

Introduction

Comment 2.1 The conceptual framework (interaction vs co-infection vs shared exposures) could be more explicitly structured. A more succinct description of uniqueness in comparison to earlier evaluations would be beneficial.

Response:

We thank the reviewer for these suggestions. We have added a sentence (Line 9-12) that explicitly distinguishes the three conceptual frameworks and clarifies why a risk factor synthesis is necessary. Regarding the uniqueness of the review relative to previous evidence, we respectfully note that this is already explicitly stated in the manuscript (Line 39-40). We have nonetheless restructured the paragraph to make this contrast more prominent.

Comment 2.2 The Introduction places little attention on translational implications (prevention techniques).

Response: We agree with the reviewer that the translational relevance was underemphasized in the Introduction. We have added a sentence to the final paragraph, to link the identification of shared risk factors to integrated control strategies. We reference to the WHO NTD Roadmap 2030, which is already discussed in the discussion and Conclusion, to ground the review's motivation at the outset.

Materials and Methods

Comment 3.1 Please justify use of fixed-effects model.

Response: We thank the reviewer for raising this point, as we agree that transparency around the methodological choice is important. We respectfully note that this was addressed extensively in our previous round of revisions in response to Reviewer #2, Major Suggestion Comment 1.6, where we provided a formal justification, detailed the distinction between a common-effect and fixed-effect model following Rice et al., and explained why a random-effects model was not adopted.

This justification is summarised in the Methods section (lines 149 – 152) and extensively described, including notation and assumptions, in Supplementary Text S4. We therefore believe the current text already addresses this concern and have maintained it accordingly.

Comment 3.2 Authors are requested to provide a clear operational definition of concurrent co-infection.

Response: We thank the reviewer for this comment and agree that providing a clear operational definition of concurrent co-infection is essential. We respectfully note that this point was addressed in our previous round of revisions following comments from Reviewer

#2 (Comment 2.8). We provided a clear working definition in in the Methods ("Outcome and exposures") and expanded the Limitations section to discuss the field-wide ambiguity. In the limitations we explicitly note that while disease-specific definitions exist in other public health contexts (WHO Guidelines for Hepatitis B or NIH Glossary for HIV), no universal standardized definition for co-infection currently exists within the context of (neglected) tropical diseases. Nevertheless, to directly address the reviewer's emphasis on concurrent co-infection, we added a clarifying sentence to the Methods (Outcome and Exposures) explicitly defining "concurrent co-infection" as the simultaneous detection of both parasites at a single time point.

Discussion / Conclusions

Comment 4.1 Clearly state that results show co-occurrence rather than causation.

Response: We thank the reviewer for this comment. Having carefully re-read the Discussion and Conclusion in preparing this response, we were unable to identify remaining causal language. The Discussion explicitly states that findings should *"be interpreted primarily as a measure of co-occurrence rather than a directional risk"* and attributes the observed association to *"shared ecological and behavioral confounding rather than immunological synergy."* The Conclusion uses careful associational language throughout.

We respectfully note that this point was raised by multiple reviewers in the previous round (Reviewer #1 Comment 1.1, Reviewer #2 Comment 2.19, and Reviewer #3 Comment 3.2) and was addressed at that stage via a thorough revision of the entire manuscript.

We are therefore confident that the current text clearly addresses this concern.

Comment 4.2 Add limitation that high heterogeneity in a fixed-effects model.

Response: We thank the reviewer for this specific point. We agree that the particular implication regarding interpretation within the inverse-variance fixed-effects framework was not explicitly stated.
We have therefore expanded the Limitations clarifying that under this framework, high heterogeneity implies the pooled estimate represents a precision-weighted average of the included study-specific effects rather than a single generalizable common effect, and that caution is warranted when applying these estimates beyond the settings represented in this review.

Comment 4.3 Shorten/specify, discussion of species comparison.

Response: We thank the reviewer for this suggestion. We have condensed the species comparison paragraph in the Discussion. We shortened the final sentence discussing biological hypotheses, removing the detail regarding the Th2-biased immune response and anemia burden, and replacing it with a more concise statement that both species share key immunological and hematological effects relevant to malaria susceptibility.
