# Supplementary material for: Shared risk factors for malaria and schistosomiasis co-infection: A systematic review and meta-analysis
Source: PLoS Negl Trop Dis. 2026 Jun 15;20(6):e0014369. doi: 10.1371/journal.pntd.0014369 (PMC13268186; doi:10.1371/journal.pntd.0014369)
Supplement: Attachment — Submitted filename: Reviewer Comments.docx [file pntd.0014369.s016.docx]

The manuscript is a decent synthesis of shared risk variables for malaria–schistosomiasis co-infection. This publication tackles a significant public health concern in NTD epidemiology. The analysis is thorough, and the methodical approach is generally strict. After a major review, the material is timely, pertinent, and ready for publishing.

However, in order to improve validity, transparency, and policy relevance, certain methodological and interpretive flaws need to be fixed.

**Minor Suggestions:**

1) Explain why trials using various diagnostic techniques were combined without making any adjustments.

2) Give age categories and the harmonization method more precise definitions.

3) For readers who are not statisticians, provide a better explanation of the terms used in "common-effects models."

4) Consider including a summary table of the main pooled effects with GRADE-style confidence and heterogeneity.

**Major Suggestions:**

5) Risk of Bias Assessment Insufficiently Integrated,

a) Discuss how methodological weaknesses (e.g., lack of clustering adjustment, unclear co-infection definitions, unmeasured confounding) may bias pooled estimates.

b) Add sensitivity analyses excluding moderate/high ROB studies.

6) Choice of Fixed-Effects Model Despite High Heterogeneity

a) The authors report very high heterogeneity in almost all pooled analyses (I² > 80%).
b) Nevertheless, they apply a fixed-effects (common-effects) model throughout.
c) Re-run primary meta-analyses using random-effects models.
d) Present both models or justify clearly why fixed-effects was retained.

**Original comments of the reviewer**

**Abstract**

1) Clarify the study objective and framework in the abstract (i.e., whether the focus is on co-infection, shared risk factors, or exposure–outcome relationships) to improve reader understanding of the scope of the review.
2) Temper the interpretation of findings by avoiding causal language and briefly acknowledging the high heterogeneity across studies, which will strengthen the scientific accuracy of the abstract.

**Introduction**

3) Make the conceptual framework clear at the outset, whether the emphasis is on interaction effects, shared exposure contexts, or actual biological co-infection.
4) By clearly outlining the shortcomings of earlier reviews and what this review specifically adds, you can strengthen the knowledge gap statement.
5) Minimize the amount of background information about specific diseases and highlight how important their interactions are to public health.
6) Make the last paragraph better by clearly outlining the goals and hypotheses.

**Materials and Methods**

7) Justify the use of a fixed-effects model or do a fresh analysis using random-effects models where there is substantial variability.
8) Describe co-infection precisely and set it apart from studies that treat one illness as an exposure and the other as a consequence.
9) Describe co-infection precisely and set it apart from studies that treat one illness as an exposure and the other as a consequence.
10) Provide more details on the handling of cluster-adjusted estimates and confounding variables.
11) Explain the rationale behind integrating studies utilizing many diagnostic methods.
12) Explain the evaluation of bias risk and its impact on interpretation.

**Results**

13) Rearrange the findings to show the conceptual difference between exposure-outcome relationships and shared risk variables.
14) Provide important pooled estimates along with heterogeneity and confidence levels in a succinct summary table.
15) Analyses with limited included studies (such as water contact) should be interpreted with caution.
16) Any sensitivity analysis results should be clearly reported, or their lack should be explained.

**Conclusions**

17) Give a succinct summary of the most reliable results.
18) Because of the significant variability in observational designs, avoid making too many generalizations.
19) Clearly state the research priorities, particularly for interventional and longitudinal studies.
20) Stress the importance of comprehensive NTD control methods.

**Discussion**

21) When assessing cross-sectional data, steer clear of causative terminology and instead focus on relationships rather than causation.
22) Incorporate risk of bias findings more directly into the evidence certainty debate.
23) Extend the conversation about the ecological context and biological plausibility of the relationship between schistosomiasis and malaria.
24) Incorporate integrated surveillance, school-based interventions, and WASH methods into the section on policy implications.

**References**

25) Several references have uneven formatting (such as different journal name abbreviations, punctuation, and italics usage); the PLOS reference style should be followed throughout the reference list.
26) Certain citations in the text should be carefully cross-checked for accuracy because they do not exactly match the reference list in terms of author order, year, or spelling.
27) A few references seem to lack comprehensive bibliographic information, such as page numbers, volume, or DOI. Whenever possible, these should be finished.
28) Verify that the reference list contains all of the references listed in the text and that there are no uncited references left.

**Grammatical Suggestions:**

|  | Original Text | Issue | Suggested Correction |
| --- | --- | --- | --- |
| 41 | literature?. | Incorrect punctuation | literature? |
| 45 | CRD42025064882) | Inconsistent ID format | Should match the earlier mentioned in the abstract ID CRD420250648824 |
| 52 | ”P. falciparum” | Smart quotes inconsistent | Use standard quotes: "P. falciparum" |
| 69 | diarrhoea | Inconsistent spelling (British vs American) | Consider using consistent spelling throughout (e.g., diarrhea) |
| 85–86 | A randomly selected 10% of full-text publications | Awkward phrasing | Ten percent of full-text publications were randomly selected |
| 96 | schistosome-malaria | Inconsistent hyphenation | Use consistently: schistosome–malaria |
| 145 | I2-statistic | Formatting | I² statistic |
| 169 | Study characteristics (no punctuation) | Heading style inconsistency | Add a colon or formatting consistent with other headings |
| 200 | lack of an independent or blinded outcome | Incomplete phrase | lack of independent or blinded outcome assessment |
| 248 | type of effect measure | Slightly ambiguous | type of effect estimate |
| 297 | model Setups | Capitalization error | model setups |
| 305 | (Y ) | Extra space | (Y) |
| 330 | however | Missing comma | however, |
| 356 | Our findings reveal that the risk profile... | Long sentence | Consider splitting for clarity |
| 418–421 | Because water contact is... | Sentence fragment | Merge with the previous sentence |
| 456–457 | co-infected vs. not co-infected | Informal style | co-infected versus non–non-co-infected |
