# Supplementary material for: Shared risk factors for malaria and schistosomiasis co-infection: A systematic review and meta-analysis
Source: PLoS Negl Trop Dis. 2026 Jun 15;20(6):e0014369. doi: 10.1371/journal.pntd.0014369 (PMC13268186; doi:10.1371/journal.pntd.0014369)
Supplement: Attachment — Submitted filename: Reviewer Comments.docx [file pntd.0014369.s018.docx]

**Original comments of the reviewer**

The clarity, organization, and methodological transparency of the revised manuscript have been significantly improved. Several major issues from the first round have been addressed by the writers, especially with regard to:

1) More precise expression of goals.
2) Better methodological explanation (subgroup analyses, search approach).
3) Extended conversation about interpretation and heterogeneity.
4) Improved correlation between results and public health significance.

**Abstract**

1) Please consider changing the language that is causal:"more frequently co-occurring" to "associated with higher odds".
2) Include a statement that acknowledges: High heterogeneity and how it affects interpretation.

**Introduction**

1) The conceptual framework (interaction vs co-infection vs shared exposures) could be more explicitly structured. A more succinct description of uniqueness in comparison to earlier evaluations would be beneficial.
2) The Introduction places little attention on translational implications (prevention techniques).

**Materials and Methods**

1) Please justify use of fixed-effects model.
2) Authors are requested to provide a clear operational definition of concurrent co-infection.

**Discussion/ Conclusions**

1) Clearly state that results show co-occurrence rather than causation.
2) Add limitation that high heterogeneity in a fixed-effects model.
3) Shorten/specify, discussion of species comparison.
